# A recurrent *SHANK1* mutation implicated in autism spectrum disorder causes autistic-like core behaviors in mice via downregulation of mGluR1-IP3R1-calcium signaling

**DOI:** 10.1038/s41380-022-01539-1

**Published:** 2022-04-06

**Authors:** Yue Qin, Yasong Du, Liqiang Chen, Yanyan Liu, Wenjing Xu, Ying Liu, Ying Li, Jing Leng, Yalan Wang, Xiao-Yong Zhang, Jianfeng Feng, Feng Zhang, Li Jin, Zilong Qiu, Xiaohong Gong, Hongyan Wang

**Affiliations:** 1 Obstetrics and Gynecology Hospital, NHC Key Laboratory of Reproduction Regulation, Shanghai Institute of Planned Parenthood Research, State Key Laboratory of Genetic Engineering at School of Life Sciences, Children’s Hospital, Fudan University, Shanghai, China; 2 Department of Child & Adolescent Psychiatry, Shanghai Mental Health Center, Shanghai Jiao Tong University School of Medicine, Shanghai, China; 3 Institute of Brain Science, Fudan University, Shanghai, China; 4 School of Integrated Chinese and Western Medicine, Institute of Integrated Chinese and Western Medicine, Anhui University of Chinese Medicine, Hefei, China; 5 Institute of Science and Technology for Brain-Inspired Intelligence, Fudan University, Shanghai, China; 6 Key Laboratory of Computational Neuroscience and Brain-Inspired Intelligence, Ministry of Education, Fudan University, Shanghai, China; 7 Shanghai Key Laboratory of Female Reproductive Endocrine Related Diseases, Institute of Metabolism and Integrative Biology, Institute of Reproduction and Development, Institutes of Biomedical Sciences, Fudan University, Shanghai, China; 8 Institute of Neuroscience, State Key Laboratory of Neuroscience, Chinese Academy of Sciences, Shanghai, China

**Keywords:** Genetics, Molecular biology, Neuroscience, Autism spectrum disorders

## Abstract

The genetic etiology and underlying mechanism of autism spectrum disorder (ASD) remain elusive. *SHANK* family genes (*SHANK1/2/3*) are well known ASD-related genes. However, little is known about how *SHANK* missense mutations contribute to ASD. Here, we aimed to clarify the molecular mechanism of and the multilevel neuropathological features induced by *Shank1* mutations in knock-in (KI) mice. In this study, by sequencing the *SHANK1* gene in a cohort of 615 ASD patients and 503 controls, we identified an ASD-specific recurrent missense mutation, c.2621 G > A (p.R874H). This mutation demonstrated strong pathogenic potential in in vitro experiments, and we generated the corresponding *Shank1* R882H-KI mice. *Shank1* R882H-KI mice displayed core symptoms of ASD, namely, social disability and repetitive behaviors, without confounding comorbidities of abnormal motor function and heightened anxiety. Brain structural changes in the frontal cortex, hippocampus and cerebellar cortex were observed in *Shank1* R882H-KI mice via structural magnetic resonance imaging. These key brain regions also showed severe and consistent downregulation of mGluR1-IP3R1-calcium signaling, which subsequently affected the release of intracellular calcium. Corresponding cellular structural and functional changes were present in *Shank1* R882H-KI mice, including decreased spine size, reduced spine density, abnormal morphology of postsynaptic densities, and impaired hippocampal long-term potentiation and basal excitatory transmission. These findings demonstrate the causative role of *SHANK1* in ASD and elucidate the underlying biological mechanism of core symptoms of ASD. We also provide a reliable model of ASD with core symptoms for future studies, such as biomarker identification and therapeutic intervention studies.

## Introduction

Autism spectrum disorder (ASD) is a lifelong neurodevelopmental disorder characterized by two core symptoms: deficits in social interaction and communication and the presence of restricted interests and repetitive behaviors [1]. The genetic etiology and underlying mechanism remain elusive, even though the heritability of ASD is as high as 70–90% [2, 3]. As major scaffolding proteins, SHANK proteins (SHANK1/2/3) individually play important roles at postsynaptic densities (PSDs) of glutamatergic synapses, where numerous pathways associated with ASD-risk genes converge [4–6]. *SHANK* family genes (*SHANK1/2/3*) are well-known ASD-related genes with multiple types of molecular defects [7], since the first report of *SHANK3* mutations in ASD patients was published in 2007 [8]. Earlier research found that deletions of *SHANK* genes account for a large percentage of *SHANK*-related ASD cases [9]. In the last decade, with the development of whole-exome sequencing (WES) and whole-genome sequencing (WGS), approximately 50% of *SHANK*-related ASD cases have been attributed to missense mutations in *SHANK* genes (https://www.sfari.org/). However, the mechanisms of ASD induced by *SHANK* missense mutations are poorly understood. To date, most *Shank-*related mouse models have been produced through disruption of entire *Shank* genes, which might not mimic the pathophysiology caused by missense mutations identified in ASD patients due to the possible gain-of-function effects produced by missense mutations or the potential genetic compensation response caused by gene knockout (KO). Although two knock-in (KI) mouse models of *Shank3* missense mutations have been reported thus far, autistic-like core behaviors are absent or mild in these mice [10, 11]. The limited research on two lines of *Shank3*-KI mice seems inconsistent with the importance of the *SHANK* family in ASD and the predominance of missense mutations in ASD-related *SHANK* variants. Therefore, further study of *SHANK* missense mutations in KI mice might aid in deciphering the heterogeneity of ASD, especially the essential mechanism related to the core symptoms.

The role of *SHANK1* in ASD has been largely ignored. *SHANK1* has been thought to be a low-risk gene for ASD based on findings that a *Shank1*-KO mouse model does not display apparent repetitive behaviors or robust social deficits [12–15] and that overexpression of *Shank1* does not alter synaptic density [16–19]. However, the genetic compensation response triggered by gene KO may ablate the expected phenotypes in KO animal models [20, 21]. The important roles of *Shank1* in synaptogenesis and synapse maturation also cannot be ignored, as overexpression of *Shank1* induces spine maturation as well as spine head enlargement [16], and knockdown of *Shank1* in vitro or KO of *Shank1* in mice results in decreased synaptic density [12, 17]. In addition, in both humans and rodent animals, *Shank1* expression is present exclusively in the brain and is particularly enriched in the cortex, hippocampus and cerebellum, which is spatiotemporally different from the expression of *Shank2* and *Shank3* [22–26] (see also the HPA RNA-seq normal tissue dataset and the Human Brain Transcriptome database). The above evidence indicates that *SHANK1* is not redundant. Notably, among *SHANK1* variant-related ASD cases, *SHANK1* missense mutations account for more than 80% of the cases (Supplementary Table 1) [18, 19, 27–29]. However, a *SHANK1* missense mutation KI mouse model has not yet been reported. Thus, generation and characterization of an ASD-related *SHANK1* missense mutation KI mouse model would help to better elucidate the pathological role of *SHANK1* in ASD.

In the present study, we first sequenced the *SHANK1* gene in a cohort of 613 ASD patients and 507 controls and found six ASD-specific missense mutations. A novel recurrent missense mutation, c.2621 G > A (p.R874H), experimentally showed the strongest pathogenic potential and was then chosen to generate a corresponding *Shank1* R882H-KI mouse model. A series of abnormalities ranging from molecular and neuronal abnormalities to brain and behavioral abnormalities were characterized in *Shank1* R882H-KI mice. Our studies clarify the molecular mechanism of and the multilevel neuropathological features induced by the *Shank1* R882H mutation in KI mice, which display the typical core symptoms of human ASD.

## Methods

Details are provided in Supplementary Methods, including study participants, mutation screening of *SHANK1* in ASD, in vitro functional characterization of mutations, as well as behavioral assays, neuroimaging, synapse morphology and molecular dissection of the constructed *Shank1* R882H-KI mice.

## Results

### Identification of six ASD-specific missense mutations in *SHANK1*

**Fig. 1:**
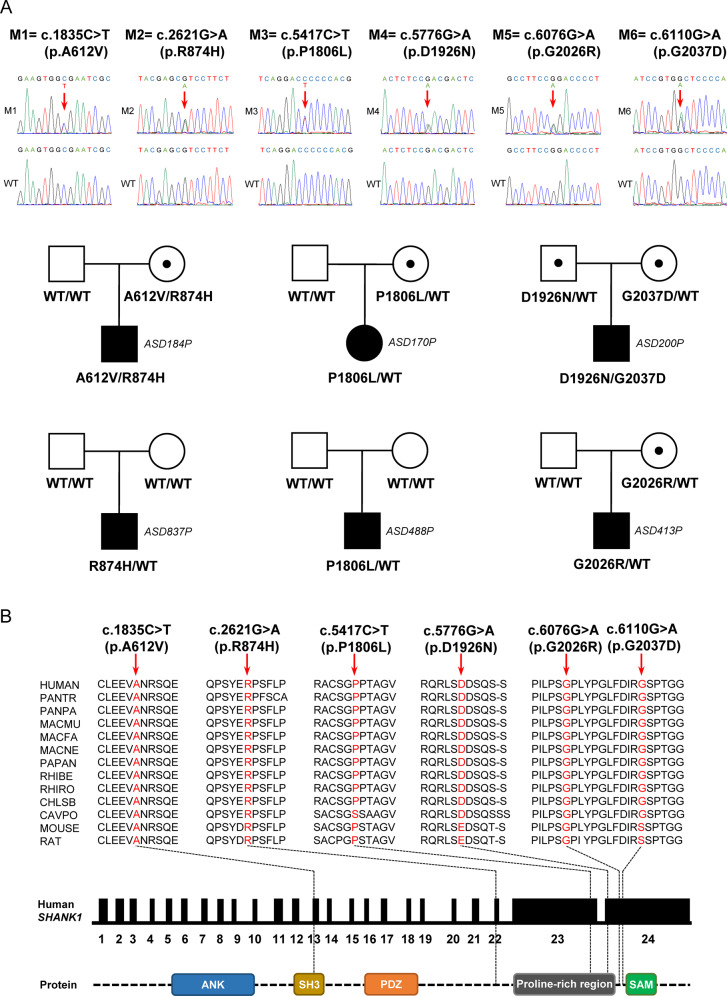
Identification of ASD-specific missense mutations in *SHANK1*. **A** Six missense mutations were identified in unrelated subjects with ASD. The position of each variant is indicated by a red arrow. Reference DNA sequences in the WT individuals are also shown. c.2621 G > A (p.R874H) was recurrent in the unrelated individuals ASD184P and ASD837P. c.5417 C > T (p.P1806L) was recurrent in the unrelated individuals ASD170P and ASD488P. Each unaffected father or mother carrier is represented by a square or circle with a black point inside. **B** The affected amino acid residues are conserved across mammalian species at different levels. Red arrows indicate the variant positions. The locations of six point mutations are noted in the schematic representation of the *SHANK1* gene and protein, whose structures have been drawn based on the information from the National Center for Biotechnology Information (NCBI) database and the UniProt Knowledgebase (UniProtKB) server. The functional domains of the SHANK1 protein are shown in colorful boxes. WT, wild-type. M, mutation. The GRCh37/hg19 coordinates are given for all variants.

Following sequencing of the coding regions and splice sites of *SHANK1* in 615 ASD patients and 503 healthy controls, six missense mutations were identified only in ASD cases, which had extremely low frequencies in 1000 Genomes Project, ExAC, gnomAD, and CMDB human population datasets (Fig. 1A, Table 1). c.2621 G > A (p.R874H) and c.5417 C > T (p.P1806L) were two recurrent missense mutations that have not been reported previously. c.2621 G > A (p.R874H) was identified in two unrelated ASD individuals: one was *de novo*, and the other was inherited from his mother, who had moderate depression and mild anxiety (Supplementary Table 2). c.5417 C > T (p.P1806L) was detected in two unrelated ASD individuals: one was *de novo*, and the other was inherited from his mother, who lacked psychiatric symptoms (Supplementary Table 2). For the ASD488P and ASD837P trios, paternity was confirmed by STR analysis (Supplementary Table 3). One ASD individual simultaneously carried two missense mutations, c.1835C > T (p.A612V) and c.2621 G > A (p.R874H), which were both inherited from his mother, who had moderate depression and mild anxiety (Supplementary Table 2). Another ASD individual simultaneously carried two missense mutations, c.5776 G > A (p.D1926N) and c.6110 G > A (p.G2037D), which were respectively inherited from his father, who had mild depression and anxiety, and his mother, who lacked psychiatric symptoms (Supplementary Table 2). c.6076 G > A (p.G2026R) was detected in one ASD individual, which was inherited from his mother (Supplementary Table 2). In total, A612V, R874H, P1806L and G2037D were first identified in patients with ASD, while D1926N and G2026R had been previously observed [18, 19]. All *SHANK1* variants identified here were heterozygous. No other types of variants, such as nonsense, frameshift or splicing-site mutations, were identified in the present cohort.

**Table 1:** Overview of the *SHANK1* variants identified in ASD subjects.

Variants						
SNP ID	rs780734174	rs143496044	rs1433344069	rs374230001	rs200040610	NA
DNA change	c.1835C > T	c.2621 G > A	c.5417 C > T	c.5776 G > A	c.6076 G > A	c.6110 G > A
AA alteration	p.A612V	p.R874H	p.P1806L	p.D1926N	p.G2026R	p.G2037D
Minor Allele Frequency in Human Population^a^						
1000 Genomes	0 (0)	2.00E-04 (1/5008)	0 (0)	2.00E-04 (1/5008)	7.99E-04 (4/5008)	0 (0)
ExAC	2.50E-05 (3/120164)	5.23E-05 (6/114684)	0 (0)	4.20E-05 (1/23824)	1.74E-04 (13/74922)	0 (0)
gnomAD	1.77E-05 (5/282358)	7.18E-05 (18/250768)	1.66E-05 (4/241264)	9.10E-05 (18/197736)	1.33E-04 (33/247704)	0 (0)
CMDB^*^	1.56E-03 (30/14861)	0 (0)	0 (0)	1.09E-03 (14/10028)	0 (0)	0 (0)
Conservation^b^						
PhyloP	3.047	4.815	0.172	0.513	1.239	0.678
PhastCons	0.999	0.989	0	0	0.819	0.039
Functional prediction						
SIFT	T	D	D	D	D	D
PolyPhen-2	D	D	B	B	B	D
MutationTaster2	H	D	H	D	D	D
CADD	D	D	T	D	T	D
DANN	T	D	T	T	T	T
Number of “deleterious”	2	5	1	3	2	4

NA means not available. AA means amino acid.

^a^Allele frequencies were estimated according to the 1000 Genomes Project Phase 3, ExAC v1.0, gnomAD v2.1 and CMDB phase I databases.

^b^PhyloP assigned positive scores to sites predicted to be conserved and negative scores to sites predicted to be fast-evolving. The closer the PhastCons value was to 1, the more likely the nucleotide was to be conserved.

^*^Allele frequency is filtered by high-quality genotypes, a standard stricter than that for minor allele count in genotypes and total number of called genotypes, as noted by the CMDB database.

D indicates “damaging” in SIFT, CADD and DANN, “possibly damaging” in PolyPhen-2 and “probably deleterious” in MutationTaster2. B, H and T indicate “benign” in PolyPhen-2, “probably harmless” in MutationTaster2 and “tolerable” in SIFT/CADD/DANN, respectively.

We defined a variant as “deleterious” if the evaluation by one functional prediction tool was “D”.

The GRCh37/hg19 coordinates are given for all variants.

The locations of these mutations are illustrated in the structural diagram of the SHANK1 protein (Fig. 1B). Based on multiple alignments among species (Fig. 1B) and scores from PhyloP and PhastCons (Table 1), the variants A612V, R874H and G2026R are evolutionarily highly conserved. To investigate the potential pathogenic effects of these variants, we performed *in silico* analyses using SIFT, PolyPhen-2, MutationTaster2, CADD and DANN tools. The vast majority of these bioinformatic tools predicted that three mutations, R874H, D1926N and G2037D, were deleterious; these mutations were classified as high-risk variants (Table 1). In particular, the variant R874H was predicted to be pathogenic by all bioinformatic tools, indicating that it had the highest probability of being deleterious.

### Impaired maturation of dendritic spines in neurons overexpressing *SHANK1* mutations in vitro

**Fig. 2:**
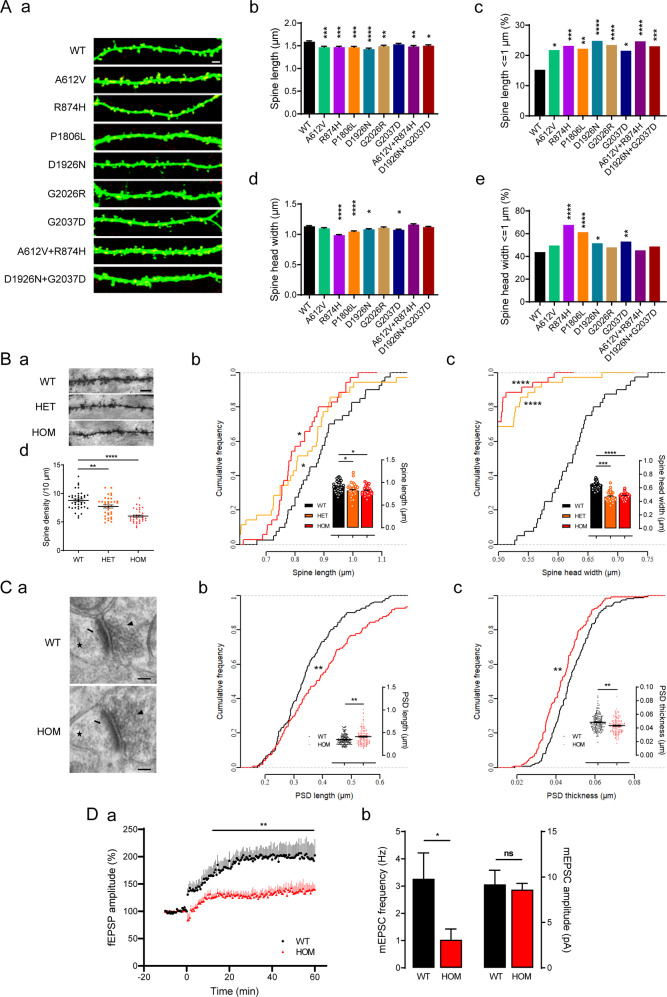
In vitro and in vivo synaptic defects in morphology and function. **A** Impaired spine maturation in cultured neurons derived from rat hippocampi affected by mutant *Shank1*. Neurons were transfected at DIV8 and examined at DIV18. **A**, **a** Examples of spines from neurons transfected with *GFP* constructs (in green) and *Shank1* constructs (in red). The scale bar is 2 µm. **A**, **b**, **d** Quantitation of spine length (µm) and head width (µm) (8–10 neurons and >650 spines examined from three to five independent experiments for each construct) (one-way ANOVA). **A**, **c**, **e** Comparisons of the percentages of spines with length ≤ 1 µm or head width ≤ 1 µm between mutant and WT *Shank1* neurons (chi-square test with Bonferroni correction). **B** Abnormal morphology of dendritic spines of CA1 neurons observed in *Shank1* R882H-KI mice with Golgi staining (35–40 neurons and >1500 spines from 3 mice per genotype). **B**, **a** Representative images of secondary dendrites. The scale bar is 5 µm. **B**, **b**, **c** Reductions in spine length and head width in R882H-KI mice, presented in cumulative frequency plots (Kruskal-Wallis test followed by Kolmogorov-Smirnov test) and bar graphs (one-way ANOVA). **B**, **d** Reduced spine density in R882H-KI mice (one-way ANOVA). **C** Ultrastructure of excitatory synapses in the hippocampus observed by TEM (>120 PSDs from 3 mice for each genotype). **C**, **a** Representative TEM images of CA1 synapses. Arrowheads, synaptic vesicles; arrows, PSDs; and stars, dendritic spines. The scale bar is 100 nm. **C**, **b**, **c** Elongated length and thinner thickness of PSDs, presented in cumulative frequency plots (Kolmogorov-Smirnov test) and scatterplots (unpaired Student’s *t*-test). **D** Perturbed glutamatergic synaptic function in *Shank1* R882H-KI mice. **D**, **a** Long-term potentiation was significantly decreased during the last 50 min of recordings in HOM mice, as is evident from the average time plot (repeated-measures ANOVA, genotype, *P* = 0.008, time, *P* < 0.0001, interaction, *P* < 0.0001; 7–8 slices from 3–4 mice for each genotype). **D**, **b** The frequency of mEPSCs was reduced, while the amplitude was not affected, in HOM mice (unpaired Student’s *t*-test; 8–10 cells from 4 mice for each genotype). All data are presented as the mean ± SE. *P < 0.05, **P < 0.01, ***P < 0.001, ****P < 0.0001 for groups compared with WT.

Shank1 is localized at postsynaptic sites, and overexpression of *Shank1* has been proven to promote spine maturation [16]. To further assess the pathogenic effects of these mutations, we performed morphological analysis of neurons in vitro. Constructs containing wild-type (WT) or mutant *Shank1* were cotransfected with *GFP* into cultured hippocampal neurons derived from rats, and then the spine neck length, spine head width and spine density were quantified (Fig. 2A). Compared with neurons overexpressing WT *Shank1*, neurons overexpressing *Shank1* mutations except G2037D showed decreases in spine length (Fig. 2A, b, c). Neurons overexpressing R874H, P1806L, D1926N or G2037D displayed significant reductions in spine width (Fig. 2A, d), of which those overexpressing R874H showed the most dramatic reductions in spine size (Fig. 2A, e). Spine density was not affected by any *Shank1* mutation (Supplementary Fig. 1A). There were no significant differences in Shank1 protein levels between these mutations and WT (*P* = 0.1426; Supplementary Fig. 1B). In summary, R874H, P1806L, and D1926N presented consistent effects on spine length and spine width. Given the high conservation and the finding that the highest score of bioinformatic pathogenicity prediction was for R874H, R874H was selected for further study in in vivo experiments to understand the mechanism of ASD induced by *SHANK1*.

### Generation of *Shank1* R882H-KI mice

KI mice with the R882H substitution (corresponding to a human R874H substitution) were generated using a CRISPR/Cas9 strategy in the C57BL/6 N strain. *Shank1* R882H-KI mice were viable and fertile and exhibited normal development. Both heterozygous (HET) and homozygous (HOM) R882H-KI mice (KI-HET and KI-HOM) had body weights similar to those of WT littermates (Supplementary Fig. 2A). Immunoblot quantification revealed no significant alterations in Shank1 protein levels in the PSD fractions derived from the whole brains of mutant mice (Supplementary Fig. 1C).

### *Shank1* R882H-KI mice displayed core behavioral features of ASD

Since the diagnostic criteria for ASD are defined behaviorally and since no biomarkers have been identified, the validity of mouse models for ASD depends strongly on their behavioral phenotypes [15]. There are two core features of ASD: persistent difficulties with social communication and social interaction, and restricted, repetitive patterns of behavior, interests, or activities. These features were assessed in *Shank1* R882H-KI mice.

### Impaired social behavior

**Fig. 3:**
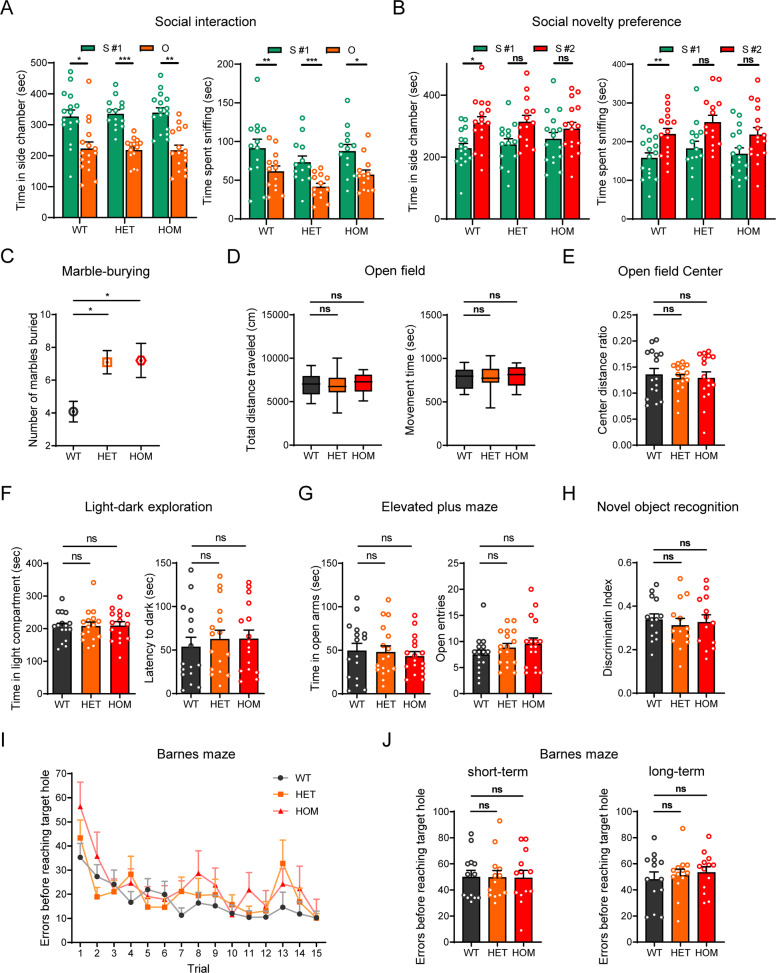
*Shank1* R882H-KI mice exhibit ASD-like core behaviors. R882H-KI mice exhibited impaired social behavior in the three-chamber social test in Experiment II (n = 14–16 for each genotype) (**A**, **B**). **A** Social interaction measured. S #1, a stranger mouse placed in one chamber. O, a novel object (inverted wire cup) placed in another chamber. **B** Social novelty preference measured. S #1, an already-investigated familiar mouse. S #2, a novel unfamiliar mouse. **C** R882H-KI mice manifest increased repetitive behaviors, as shown by burying of more marbles in the MB test (n = 20–25 for each genotype). **D** R882H-KI mice showed normal exploratory locomotion in the OF test (n = 16 for each genotype). No anxiety-like behavior was exhibited by R882H-KI mice with face validity, as shown by the center distance ratio in the OF test (**E**), the time in the light compartment in the LD exploration (**F**) (n = 16 for each genotype), and the time and entries in open arms in the EPM test (**G**) (n = 16–17 for each genotype). **H** No deficits in recognition memory in R882H-KI mice, assessed by NOR (n = 13–14 for each genotype). **I** Normal performance of spatial learning in the BM (n = 13 for each genotype), measured by the number of errors made before reaching the target hole (genotype, *P* = 0.1921, trial, *P* < 0.0001, interaction, *P* = 0.7528). **J** No deficits in short-term (Day 5) or long-term (Day 12) spatial memory were observed in R882H-KI mice, as indicated by the number of errors made before reaching the target hole. Paired Student’s *t*-test for **A**, **B**. One-way ANOVA for **C**–**H**, **J**. Repeated-measures ANOVA for **I**. All data are presented as the mean ± SE. ns, no significance, *P < 0.05, **P < 0.01, ***P < 0.001 for the groups compared with WT.

Both social interaction and social novelty preference were examined with three-chamber social test and determined based on consistent results by chamber time and sniffing time [30]. In the social interaction test, mice were given the choice to interact with an inanimate object or a stranger mouse. KI-HET and KI-HOM mice presented normal social interaction, comparable to WT mice, spending significantly more time in the chamber with the stranger mouse than in that with the object in Experiment II (Fig. 3A). Simultaneously, there existed differences in the sniffing time spent with the object or the stranger mouse in KI-HET and KI-HOM mice (Fig. 3A). In addition, the results of chamber time and sniffing time in Experiment I were inconsistent (Supplementary Fig. 2A). Consequently, the social interaction of R882H-KI mice was not impaired in our experiments. In the test of social novelty preference, WT littermates performed normally, spending more time in the chamber containing the novel mouse (stranger #2) than in that containing the familiar mouse (stranger #1) (Fig. 3B). However, neither KI-HET nor KI-HOM mice showed differences in the time spent in the two chambers (Fig. 3B), exhibiting impairments in social novelty preference. Assessment of direct sniffing interaction with the familiar or novel mouse, another sensitive measure of social novelty preference, also supported the disability of social novelty preference in R882H-KI mice. Compared with WT mice, neither KI-HET nor KI-HOM mice showed significantly more sniffing time with stranger #2 than with stranger #1 (Fig. 3B). As a within-task control for levels of general exploratory locomotion, entries into the left and right side chambers were measured, but the entries did not differ for the three genotypes (Supplementary Fig. 2C). Collectively, the social behavior of KI mice was impaired, manifesting in the aspect of social novelty preference.

### Increased repetitive behaviors

The marble-burying (MB) test has been proven to be a reliable method for measurement of repetitive behaviors in mouse models [31, 32]. The extent of repetitive behaviors is reflected by the number of buried marbles. In this assay, marble-burying performance significantly differed among the three genotypes (Fig. 3C). *Post hoc* analysis showed that more marbles were buried by both KI-HET and KI-HOM mice than by WT littermate controls. This meant that R882H mutants exhibited increased repetitive behaviors.

### Normal locomotor ability and no anxiety-like behavior

Comorbid anxiety symptoms and motor abnormalities appear in approximately 42–56 and 79% of ASD cases, respectively [33]. With regard to mouse behaviors, motor abnormalities or heightened anxiety could affect performance and confound interpretations of results related to the core phenotypes [13, 31, 34, 35]. To assess locomotor ability and anxiety-like behavior, we performed an open-field (OF), light-dark (LD) exploration and elevated plus maze (EPM) test. The R882H-KI mice showed normal locomotor activity, as measured by total distance traveled and movement time in the OF (Fig. 3D). This was consistent with the results for the numbers of entries in three-chamber tests (Supplementary Fig. 2C), the numbers of transitions between two compartments in the LD test (Supplementary Fig. 2D), and the numbers of total entries into each arm in the EPM test for three genotypes (Supplementary Fig. 2E), all of which served as within-task controls for the levels of general exploratory locomotion. Anxiety-like behaviors were not detected in mutant mice, as assessed by the central tendency in the OF test (Fig. 3E), the time spent in the light compartment in the LD test (Fig. 3F) as well as the time spent in open arms and the entries into open arms in the EPM test (Fig. 3G), compared to WT littermates. These data provide further evidence to support the findings that impaired sociability and repetitive behaviors in R882H-KI mice are intrinsic and unaffected by comorbidities such as abnormal motor function and heightened anxiety.

### No learning or memory deficits

In addition to the core symptoms, a number of individuals with ASD have atypical cognitive profiles [33]. Here, in mice, recognition memory was evaluated by the novel object recognition (NOR) assay [36]. Spatial learning and memory were assessed with the Barnes maze (BM) task, which assesses a mouse’s ability to learn the location of a target zone with the use of visual cues [37]. R882H-KI mice showed similar discrimination scores for the novel object to WT mice, indicating that object recognition memory were not affected in mutant mice (Fig. 3H). In BM, both mutant and WT mice were able to successfully learn the task, as evidenced by similar decreasing trends in the numbers of total errors before finding the escape box over the course of 15 trials (Fig. 3I) and supported by another parameter, latency (Supplementary Fig. 2F). On day 5, there was no difference in the number of total errors or the percentage of time spent in the target quadrant between mutant and WT mice (Fig. 3J and Supplementary Fig. 2G), indicating that mutant mice had normal short-term retention. On day 12, mutant mice displayed more errors and less quadrant time than WT mice, reflecting the trends of impairment in long-term retention, but these differences did not reach statistical significance (Fig. 3J and Supplementary Fig. 2G). Overall, no obvious impairment of spatial learning or memory was observed in mutant mice.

### Brain structural changes in *Shank1* R882H-KI mice focused on the frontal cortex, hippocampus and cerebellar cortex

**Fig. 4:**
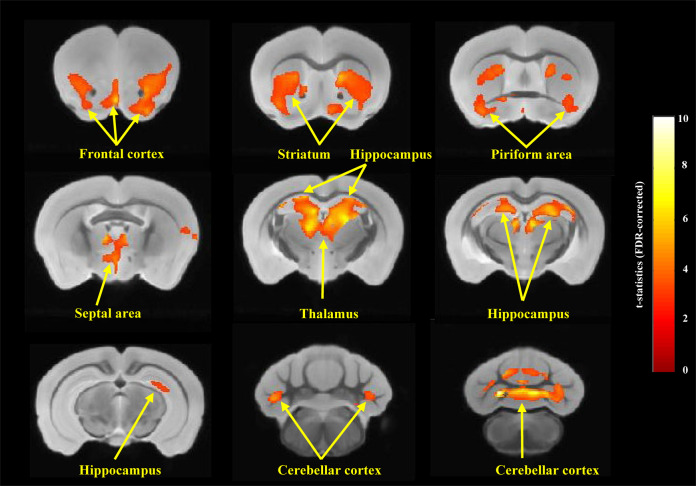
Prominent changes in GMV focusing in frontal, hippocampal and cerebellar regions of *Shank1* R882H-KI HOM mice. Structural MRI with T2-weighted images revealed the GMVs of multiple brain regions significantly increased in *Shank1* R882H-KI HOM mice compared with WT mice. Of these regions, the frontal cortex, hippocampus and cerebellar cortex showed dramatically increased GMVs dramatically increased GMV in frontal cortex (FC), hippocampus (HP) and cerebellar cortex (CBC) in *Shank1* R882H-KI HOM mice. Piriform area has the frontal and temporal components. Septal area is the part of the frontal lobe. Voxel-based morphometry (VBM) analysis of brain coronal sections on an averaged MRI template by comparing *Shank1* R882H-KI HOM mice (n = 8) with WT mice (n = 9) (*P* < 0.05, clusters >200 voxels, uncorrected, then followed by FDR correction with significance of 0.05).

To investigate potential neuroanatomical abnormalities underlying ASD-related core behaviors in R882H-KI mice, the brains of WT mice and KI-HOM mice were imaged using in vivo structural magnetic resonance imaging (sMRI). Voxel-based morphometry (VBM) was applied to measure gray matter volume (GMV) differences between the two groups. Significant differences in GMV were obtained in the frontal cortex (including septal area), striatum, piriform area, thalamus, hippocampus and cerebellar cortex, where KI-HOM mice revealed a notable increase compared with WT mice (Fig. 4). Of these regions, the frontal cortex, hippocampus and cerebellar cortex were regions where murine *Shank1* is the most highly expressed [23–25] and were also implicated in ASD in previous neuroimaging studies on humans and mice. The discovery of structural changes in KI-HOM mice further supported the pathological effects of these regions in ASD. Therefore, these three regions were selected as key brain regions for further exploration.

### Synaptic structural and functional anomalies in *Shank1* R882H-KI mice

At the cellular level, we examined spine morphology and spine density in hippocampal CA1 pyramidal neurons by Golgi staining. Similar to the morphological changes in vitro, reduced spine width and length were observed in mutant mice. A significantly shorter spine length was found in R882H mutant mice than in WT littermates, which was evident by the shift of the cumulative probability to the left (0.82 ± 0.03 μm and 0.81 ± 0.02 μm in HET and HOM, respectively, versus 0.89 ± 0.02 μm in WT; Fig. 2B, a, b). R882H-KI mice showed a pronounced reduction in spine width, with a dramatic shift to the upper left quadrant in cumulative probability plots (0.47 ± 0.01 μm and 0.48 ± 0.01 μm in HET and HOM, respectively, versus 0.62 ± 0.01 μm in WT, Fig. 2B, a, c). In terms of spine density, R882H-KI mutants displayed declines of 12.2% and 31.4% in HET and HOM mice, respectively, compared with their WT littermates (from 8.78 ± 0.25 in WT to 7.71 ± 0.29 in HET and 6.02 ± 0.20 in HOM per 10 μm; Fig. 2B, d), which was different from the result of no change in cultured neurons in vitro.

We compared the ultrastructure of glutamatergic synapses in hippocampal CA1 neurons between WT and KI-HOM mice using electron microscopy (Fig. 2C). PSD length was increased by 17% in KI-HOM mice (from 0.35 ± 0.01 μm in WT to 0.41 ± 0.02 μm in HOM; Fig. 2C, a, b). A reduction of 10% was observed in the thickness of the PSDs of KI-HOM mice relative to controls (from 0.048 ± 0.001 μm in WT to 0.043 ± 0.001 μm in HOM; Fig. 2C, a, c).

We performed electrophysiological recordings of the hippocampus to assess synaptic function. Extracellular recordings at SC-CA1 synapses showed that long-term potential (LTP) induced by theta burst stimulation (TBS) of the SCs was severely impaired in KI-HOM mice from the induction period to the maintenance period; the amplitude of field excitatory postsynaptic potentials (fEPSPs) was 25.1% lower in KI-HOM mice than in WT mice (Fig. 2D, a). Basal synaptic transmission at CA1 pyramidal neurons was assessed by mEPSCs using whole-cell patch-clamp recordings. A significantly decrease of mEPSCs in the frequency, but not the amplitude, was observed in KI-HOM mice compared with controls (Fig. 2D, b), indicating weakened basal excitatory transmission.

### Dysfunction of mGluR1-IP3R1-calcium signaling in *Shank1* R882H-KI mice

#### Downregulation of mGluR1-IP3R1 signaling

Shank proteins are described as the “master regulators” of glutamatergic synapses, as they function as core components of the PSD to modulate synaptic structure and function. We tried to illuminate the molecular basis responsible for the aberrant synaptic structure and function observed in R882H-KI mice. First, we performed quantitative proteomic analysis of PSDs from the hippocampus through LC-MS/MS. In total, 2081 proteins were identified, of which 472 proteins (22.7%) were differentially expressed in KI-HOM versus WT mice. The glutamatergic synapse was the most significant term among the 9 enriched terms in KEGG pathway analysis (Supplementary Fig. 3B). In this enriched pathway, there were 14 proteins located in postsynapses, which were ionotropic glutamate receptors (NMDAR subunits GluN1/N2A and AMPAR subunits GluA1/A2/A4), metabotropic glutamate receptor (mGluR1), all members of the Homer family (Homer1/2/3), PSD-95, IP3R, extracellular-signal-regulated kinase (ERK) 1/2 and calcineurin (Ppp3ca/3cb) (Supplementary Table 4). All of them have direct or indirect interactions with SHANK1. In RNA-Seq analysis of mRNAs in the hippocampus in KI-HOM and WT mice, 23718 genes were identified. No differences were found for any of the aforementioned molecules, and only 14 genes showed significant changes (Supplementary Table 5). However, the functions of these genes are unknown, or the genes are not directly related to ASD. No terms were enriched in KEGG pathway analysis (Supplementary Fig. 3A).

**Fig. 5:**
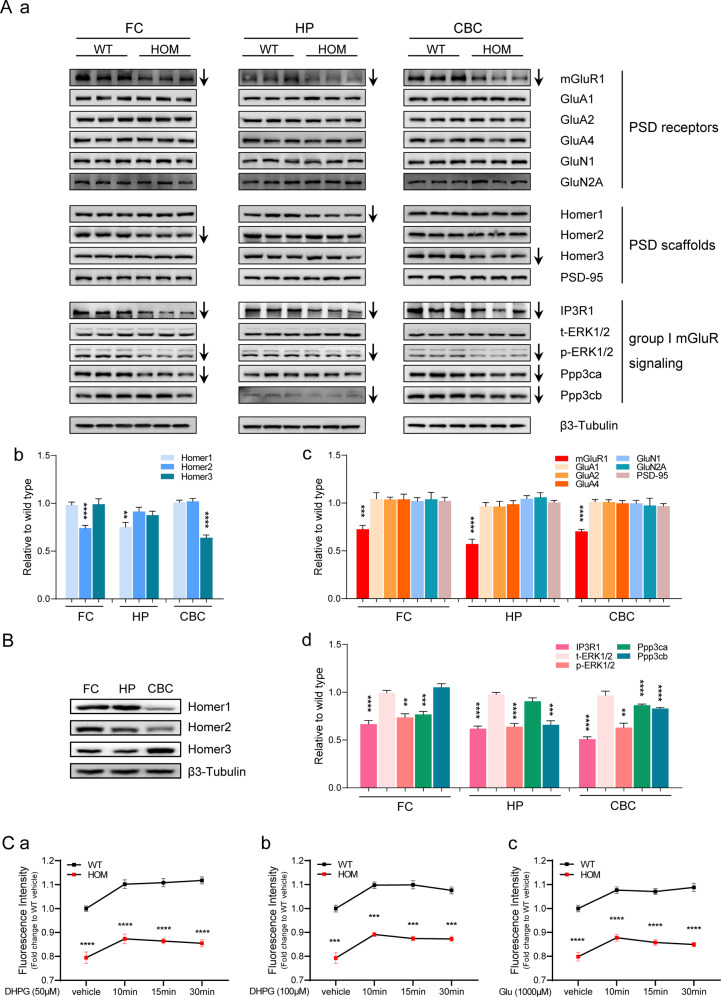
Biochemical changes in PSD fractions from multiple brain regions and diminished release of intracellular calcium in *Shank1* R882H-KI HOM mice. **A** Immunoblot analyses of PSD fractions from the frontal cortex (FC), hippocampus (HP) and cerebellar cortex (CBC) in WT and *Shank1* R882H-KI HOM mice. **A**, **a** Representative results for the indicated proteins. Each lane was loaded with protein samples from an individual mouse, with β3-Tubulin as a loading control, and normalized to WT levels (n = 3 mice per group). **A**, **b** The levels of Homer family proteins were significantly reduced in three brain regions of R882H-KI mice. A specific member of the Homer family proteins was downregulated in each brain region, with Homer1 downregulated in the HP, Homer2 downregulated in the FC and Homer3 downregulated in the CBC. **A**, **c** Only mGluR1 protein levels were decreased in the three regions of R882H-KI mice. No changes in AMPAR (GluA1, GluA2 and GluA4), NMDAR (GluN1 and GluN2A) or PSD-95 were observed. **A**, **d** The protein levels of IP3R1 and the calcium signaling-associated molecules p-ERK1/2, Ppp3ca and Ppp3cb were also reduced in three brain regions of R882H-KI mice, while t-ERK1/2 levels in these mice were comparable to those in WT mice. The legend p-ERK1/2 represents the ratio of p-ERK1/2 to t-ERK1/2. All data were obtained from three pairs of mice tested at least in duplicate and are presented as the mean ± SE. *P < 0.05, **P < 0.01, ***P < 0.001, ****P < 0.0001 for HOM groups compared with WT, by unpaired Student’s *t*-test. B Protein levels of Homer1/2/3 in PSD fractions from three brain regions. Homer1 is highly expressed in the HP, Homer2 is highly expressed in the FC, and Homer3 is highly expressed in the CBC in WT mice. The bands are representative of two or three independent experiments. For the statistical results, see Supplementary Fig. 6C. **C** Flow cytometry was used to determine the concentrations of intracellular calcium in the hippocampal neurons of WT and *Shank1* R882H-KI HOM mice (n = 5 for each genotype). **C**, **a**, **b** Effects of stimulation with 50 µM and 100 µM DHPG on the intracellular calcium concentration ([Ca^2+^]_i_). Hippocampal neurons in mice of both genotypes exhibited elevated [Ca^2+^]_i_ (WT: **** for 10 min, 15 min and 30 min vs. vehicle; HOM: *** for 10 min, ** for 15 min and 30 min vs. vehicle). At each time point, [Ca^2+^]_i_ in HOM mice was always lower than that in WT mice. **C**, **c** Effect of stimulation with 100 µM glutamate (Glu) on [Ca^2+^]_i_. Hippocampal neurons in mice of both genotypes exhibited elevated [Ca^2+^]_i_ (WT: **** for 10 min, 15 min and 30 min vs. vehicle; HOM: **** for 10 min and 15 min, * for 30 min vs. vehicle). At each time point, [Ca^2+^]i in HOM mice was always lower than that in WT mice. The value presented is the mean fluorescence intensity induced by Fluo-4. All data were obtained from five independent experiments and are presented as the mean ± SE. *P < 0.05, **P < 0.01, ***P < 0.001, ****P < 0.0001 for HOM groups compared with WT, by two-way ANOVA.

Next, we validated the above 14 differentially expressed proteins in KI-HOM mice and WT littermates by immunoblotting (Fig. 5A). PSD fractions from the frontal cortex, hippocampus and cerebellar cortex, whose GMVs were significantly altered in KI-HOM mice, were chosen for analyses. Among the 6 detected membrane receptors, only mGluR1 was downregulated at the protein level, while NMDAR and AMPAR subunits **(**GluN1/N2A and GluA1/GluA2/GluA4) remained unchanged in the three brain regions (Fig. 5A, a and c). mGluR1 was downregulated by up to nearly 50% (−26% in the frontal cortex, −42.6% in the hippocampus and −29.4% in the cerebellar cortex). The consistent downregulation of mGluR1 in three regions provides robust evidence of dysfunction of metabotropic glutamate receptors in mutant mice. mGluR1 is anchored to the PSD through its interaction with Homer, which directly binds to Shanks. The protein levels of Homer1, Homer2, and Homer3 were highest in the hippocampus, frontal cortex and cerebellar cortex, respectively (Fig. 5B and Supplementary Fig. 3C). Interestingly, we found that the protein levels of Homer1, Homer2 and Homer3 showed the strongest downregulation in the specific brain regions that had the highest expression levels among the three tested brain regions. A decrease of 25% was observed for Homer1 in the hippocampus, a decrease of 30.6% was observed for Homer2 in the frontal cortex, and a decrease of 34.4% was observed for Homer3 in the cerebellar cortex (Fig. 5A, a, b). The comprehensive and severe downregulation of Homer protein subtypes expression in mutant mice indicates that the effect of *Shank1* R882H on Homer proteins is general and subtype-specific. IP3R1, tethered to Shanks by Homer, is an important downstream target of mGluR1. Significant reductions of up to 50% in IP3R1 levels were observed in the three brain regions (−30.8% in the frontal cortex, −35% in the hippocampus and −50.0% in the cerebellar cortex) (Fig. 5A, a, d). Phosphorylated ERK1/2, a downstream effector of mGluR1 and IP3R1, showed reductions of 24.6% in the frontal cortex, 35.4% in the hippocampus and 37.0% in the cerebellar cortex (Fig. 5A, a, d). Calcineurin is a calcium-dependent phosphatase that plays a critical role in synaptic plasticity. Ppp3ca, a subunit of calcineurin, showed reductions of 23.3% in the frontal cortex and 13.6% in the cerebellar cortex, while another subunit of calcineurin, Ppp3cb, showed reductions of 33.3% in the hippocampus and 17.3% in the cerebellar cortex (Fig. 5A, a, d). There were no changes in PSD-95 protein levels in the three brain regions (Fig. 5A, a, c).

#### Impairment of intracellular release of calcium

IP3R1 is an intracellular calcium channel that mediates intracellular calcium release from ER calcium storage by binding with and being activated by IP3 generated through mGluR1 [38]. Since mGluR1 and IP3R1 levels were diminished in R882H-KI mice, we speculated that the amount of calcium released from the ER might have been lessened, affecting the concentration of neuronal cytosolic calcium [39]. To test this hypothesis, we measured the calcium transients in the hippocampus stimulated by DHPG, a specific group I metabotropic receptor agonist. DHPG-induced calcium transients in R882H-KI mice were significantly lower (by more than 20%) than those in WT mice (50 µM and 100 µM, Fig. 5C, a, b), indicating impaired mGluR1-IP3R1-mediated intracellular release of calcium in KI mice. Next, we tested whether glutamate-induced intracellular calcium concentration ([Ca^2+^]_i_) is affected by aberrant mGluR1-IP3R1 signaling in KI mice. The concentration of glutamate was within the range used for physiological stimulation (from low micromolar concentrations up to 1 mM) to simulate the physiological state during neurotransmission [40]. Effective responses to glutamate were obtained in hippocampal cells from both KI mice and WT controls. The [Ca^2+^]_i_ was elevated markedly at 10 min, and the elevation lasted for 30 min (Fig. 5C, c). However, the [Ca^2+^]_i_ was always significantly lower in KI mice than in WT mice (approximately 20% lower), indicating that glutamate-induced [Ca^2+^]_i_ was disturbed in mutant mice.

Molecular investigation shows that the *Shank1* R882H substitution specifically affects mGluR1-IP3R1-calcium signaling, leading to a decrease in the intracellular calcium concentration. Combined with the corresponding cellular and brain imaging changes in the same regions, these findings provide reasonable mechanistic explanations for the behavioral abnormalities observed in *Shank1* R882H-KI mice.

## Discussion

In the present study, the recurrent missense mutation c.2621 G > A (p.R874H) of *SHANK1* was identified in ASD cases for the first time, and the underlying neurological and molecular mechanisms were illuminated. This variant was located at a highly conserved region near the PRO domain of *SHANK1*. The R882H-KI mice demonstrated core symptoms of ASD, namely, social disability and repetitive behaviors, without anxiety-like behavior, locomotor abnormalities, or learning and memory deficits. We found structural changes in the frontal cortex, hippocampus and cerebellar cortex in KI mice via sMRI, which were also brain regions with highly expressed murine *Shank1*. KI mice demonstrated abnormal cellular structure and function with decreased spine length and width, reduced spine density, PSD morphological changes, impaired hippocampal LTP, and weakened basal excitatory transmission. Importantly, significant downregulation of mGluR1-IP3R1-calcium signaling in specific brain regions of KI mice was first recognized to be exclusively related to ASD core symptoms. To the best of our knowledge, among all the KI mice with ASD-related missense mutations, *Shank1* R882H mice are the only ASD mouse model that purely manifests two core symptoms of ASD unaccompanied by comorbidities such as abnormal motor function and heightened anxiety. Our study illuminates the vital role of mGluR1-IP3R1-calcium signaling in the pathogenesis of ASD with typical core symptoms for the first time and provides a reliable ASD mouse model for future studies.

The KI mouse model of *Shank1* R882H displays two ASD core symptoms with no confounding comorbidities and precisely demonstrates the causative relationship of the *SHANK1* gene with ASD. In contrast, *Shank1*-KO mice display mainly ASD-nonspecific symptoms with no typical or consistent core symptoms and can thus hardly be used to draw the certain conclusions described above [12–15]. Previous studies have found that ASD-associated social and repetitive behaviors are influenced by abnormal locomotion or anxiety-like behavior in mice. For example, low social interaction or social novelty preference could be partially attributed to low exploratory activity and/or high anxiety-like behavior in mice, thus rendering social assay data meaningless [13, 34]. Enhanced repetitive performance in marble-burying could also be affected by exploratory activity [31, 35]. A series of behavioral tests showed that R882H-KI mice are characterized by ASD core symptoms with no confounding comorbidities of heightened anxiety and abnormal locomotion. Among all of the summarized ASD-related KI mice (Supplementary Table 6), our established *Shank1* R882H model, together with the *Slc6a4* G56A model, manifests ASD core symptoms without confounding comorbidities. However, it should be noted that the increased repetitive behaviors in the *Slc6a4* G56A model are totally different from those in the *Shank1* R882H model. Specifically, restricted and repetitive behaviors (RRBs) in ASD fall into two categories: higher-order and lower-order RRBs [41–44]. Of these, only higher-order RRBs are unique to ASD, whereas lower-order RRBs are observed in many developmental disorders [45, 46]. *Shank1* R882H mice exhibited increased repetitive behaviors in the marble-burying task, which serves as a test for higher-order RRBs in mouse models [31, 47], while Slc6a4 G56A mice showed weakly increased lower-order RRBs without exhibiting abnormal higher-order RRBs [48] (Supplementary Table 6). Therefore, *Shank1* R882H mice are an appropriate model for pathological or therapeutic studies of ASD core symptoms.

This study is the first to emphasize that mGluR1-IP3R1-calcium signaling is dramatically downregulated in ASD and is especially related to ASD core symptoms. SHANK indirectly interacts with glutamate receptors via different proteins at defined functional domains: AMPARs via GRIP at the SH3 domain of SHANK, NMDARs via the PSD-95/GKAP complex at the PDZ domain and mGluRs via Homer at the PRO domain. Specifically, we determine that mGluR1-IP3R1 signaling is comprehensively and significantly dampened in brain regions of frontal cortex, hippocampus and cerebellar cortex in *Shank1* R882H-KI mice. These three brain regions are the regions in which *Shank1* is highly expressed, and structural alterations in these regions were also highlighted by sMRI scanning. Unlike *Shank1* R882H-KI mice, *Shank*-KO mice demonstrate the involvement of ionotropic glutamate receptors (NMDARs and AMPARs) in the pathogenesis of ASD, and the roles of metabotropic glutamate receptors (mGluRs) in ASD in these mice seem ambiguous or unimportant. No changes in glutamate receptors have been observed in *Shank1*-KO mice [12], opposite changes in NMDAR function have been observed in *Shank2-*KO mice [49, 50] and downregulations of NMDARs and AMPARs have been observed in *Shank3*-KO mice [25, 51–53]. Recently, mGluR5 and mGluR5-mediated signaling were found to be altered in *Shank3*-KO mice [54, 55]. The differences in molecular changes between *Shank1*-KI and *Shank1-*KO mice would lead to different pathological processes and totally different outcomes. Here, the *Shank1* R882H-KI mouse model precisely illuminates the relationship between ASD core symptoms and the corresponding molecular changes. Our study sheds light on the irreplaceable importance of KI mouse models bearing ASD-related missense mutations for investigating and better understanding the underlying mechanism of ASD with clinical heterogeneity.

The significant downregulation of mGluR1-IP3R1-calcium signaling is highlighted to clarify the molecular mechanisms underlying the core symptoms of ASD. The fact that mGluR1 is the only altered glutamate receptor in R882H-KI mice indicates its unique and fundamental role in the pathogenesis of ASD. It has been demonstrated that group I mGluRs increase neuronal excitability [56–58], and mice deficient in mGluR1 display impaired synaptic plasticity, spatial learning deficits and severe motor coordination deficits [59, 60]. Functionally, activation of mGluR1 upon glutamate binding leads to the production of IP3, which binds to IP3R1 on the ER and subsequently triggers calcium release from the ER, thereby contributing to calcium signaling in neurons [61]. A wide range of neuronal processes, such as synaptic plasticity, neuronal excitability and neural circuit regulation, are under the control of cytosolic calcium signals [62] through the activation of multiple downstream signaling cascades, such as ERK signaling [63–65] and calcineurin signaling [66]. In our study, the levels of cytosolic calcium, downstream p-ERK1/2 (representing ERK1/2 activity) and Ppp3ca/Ppp3cb (catalytic subunit of calcineurin) were decreased in multiple brain regions. Therefore, downregulation of mGluR1-IP3R1-calcium signaling could explain the impaired synaptic plasticity and reduced basal excitatory transmission in *Shank1* R882H-KI mice and finally lead to the core behavioral disability of ASD. Moreover, a number of human genetics studies have identified multiple rare variants of HOMER1 and IP3R1, the core genes in the mGluR-IP3R pathway, in ASD patients [67–70], supporting the importance of this pathway in the development of ASD.

Among the series of molecular changes triggered by R874H, changes in HOMER proteins are impressive. SHANK1 contains multiple domains, and HOMER interacts with SHANK1 at the PRO domain, which has been largely overlooked compared with other massively studied domains, such as the SPN, ANK and PDZ domains [18, 50–52, 71, 72]. However, the majority of *SHANK1* variants reported so far are located in or near the PRO domain, and R874H is also located in a highly conserved region near the PRO domain (Supplementary Fig. 4), suggesting the critical role of the PRO domain in ASD. In our study, Homer proteins (Homer1/2/3) showed subtype-specific reductions in different brain regions. Each of the focused brain regions in KI mice showed significant impairment of a Homer subtype. The most strongly downregulated Homer subtype in a specific brain region in KI mice was usually the most highly expressed Homer in that region in WT mice. As Shank and Homer are major determinants of the sizes of dendritic spines and PSDs through forming a high-order polymerized complex [16, 73], the abnormal structures of dendritic spines and PSDs in the *Shank1* R882H-KI mouse model could be explained by decreases in Homer levels. Our findings indicate that comprehensive and severe aberrations in Homer proteins in key brain regions of *Shank1* R882H-KI mice lead to subsequent changes in other interacting proteins, profoundly affect the structure and function of postsynaptic neurons, and thereby contribute to the emergence of core symptoms of ASD.

Neuroimaging studies provide important insights into the neurological basis for ASD and the results of these studies can be leveraged to aid ASD diagnosis [74–76]. sMRI scans in *Shank1* R882H-KI mice revealed that the main ASD core symptom-related brain regions are the frontal cortex, hippocampus and cerebellar cortex, emphasizing that the neurological changes in these regions are critical for ASD and connecting the pathological process from genes to behaviors. These three regions are the most consistently affected regions both in ASD patients [77–83] and among twenty-six different ASD mouse models [84]. The observed correlations between structural impairments of these three brain regions and social disability or repetitive behaviors in ASD patients or in different ASD mouse models are also concordant with our findings. On the one hand, the GMV in frontal or cerebellar cortex has been found to be positively correlated with the severity of core symptoms in some autistic patients [82, 85]. On the other hand, the hippocampal or cerebellar GMV shows positive correlation with repetitive behaviors in BTBR mice [86]. Increased hippocampal GMV is more identified in mouse models with poor social performance than those with normal/mixed social activities [84]. These findings provide strong evidence for the involvement of the three brain regions in core autistic behaviors observed in our R882H-KI mouse model. Moreover, the consistent molecular changes in these regions further support their important roles in the pathogenesis of ASD. Interestingly, altered volumes were also found in the striatum, thalamus and piriform area, consistent with previous reports that abnormalities of the above regions were implicated in the clinical core profiles of ASD. In individuals with ASD, increased GMV in the striatum and thalamus were commonly observed from multiple independent cohorts [82, 87–92]. Volume changes in the striatum were repeatedly reported to be associated positively with repetitive behaviors in ASD [82, 87, 89, 92]. Multiple lines of research have reported thalamic abnormalities are related to both social impairments and repetitive behaviors, although the relationship remains to be further determined [93–95]. Despite lack of evidence in structural relation, the piriform area has been recently functionally associated with ASD-related social behaviors [96, 97]. Notably, all the regions identified by sMRI, except piriform area, are key components of the circuits mediating the expression of social and repetitive behaviors [97–99], further indicating their close relation to ASD.

Taken together, our findings emphasize the absolute necessity of KI mouse models carrying the possible etiological human mutations to understand the pathogenesis of ASD. *Shank1* R882H-KI mice, which exhibit two core symptoms of ASD without confounding comorbidities, provide a reliable model of ASD for future diverse studies, such as biomarker identification and therapeutic intervention studies. Identification of the role of mGluR1-IP3R1-calcium signaling in ASD reveals the molecular mechanism underlying the process from abnormal cellular structure and function to autistic-like core behaviors in *Shank1* R882H mice, which might serve as potential targets for pharmaceutical intervention.

## Supplementary information

Supplementary information
