# Correction: A recurrent *SHANK1* mutation implicated in autism spectrum disorder causes autistic-like core behaviors in mice via downregulation of mGluR1-IP3R1-calcium signaling

**DOI:** 10.1038/s41380-022-01605-8

**Published:** 2022-05-10

**Authors:** Yue Qin, Yasong Du, Liqiang Chen, Yanyan Liu, Wenjing Xu, Ying Liu, Ying Li, Jing Leng, Yalan Wang, Xiao-Yong Zhang, Jianfeng Feng, Feng Zhang, Li Jin, Zilong Qiu, Xiaohong Gong, Hongyan Wang

**Affiliations:** 1 Obstetrics and Gynecology Hospital, NHC Key Laboratory of Reproduction Regulation, Shanghai Institute of Planned Parenthood Research, State Key Laboratory of Genetic Engineering at School of Life Sciences, Children’s Hospital, Fudan University, Shanghai, China; 2 Department of Child & Adolescent Psychiatry, Shanghai Mental Health Center, Shanghai Jiao Tong University School of Medicine, Shanghai, China; 3 Institute of Brain Science, Fudan University, Shanghai, China; 4 School of Integrated Chinese and Western Medicine, Institute of Integrated Chinese and Western Medicine, Anhui University of Chinese Medicine, Hefei, China; 5 Institute of Science and Technology for Brain-Inspired Intelligence, Fudan University, Shanghai, China; 6 Key Laboratory of Computational Neuroscience and Brain-Inspired Intelligence, Ministry of Education, Fudan University, Shanghai, China; 7 Shanghai Key Laboratory of Female Reproductive Endocrine Related Diseases, Institute of Metabolism and Integrative Biology, Institute of Reproduction and Development, Institutes of Biomedical Sciences, Fudan University, Shanghai, China; 8 Institute of Neuroscience, State Key Laboratory of Neuroscience, Chinese Academy of Sciences, Shanghai, China

**Keywords:** Genetics, Molecular biology, Neuroscience, Autism spectrum disorders

Correction to: *Molecular Psychiatry* 10.1038/s41380-022-01539-1, published online 06 April 2022

The article “A recurrent SHANK1 mutation implicated in autism spectrum disorder causes autistic-like core behaviors in mice via downregulation of mGluR1-IP3R1-calcium signaling”, written by Yue Qin, Yasong Du, Liqiang Chen, Yanyan Liu, Wenjing Xu, Ying Liu, Ying Li, Jing Leng, Yalan Wang, Xiao-Yong Zhang, Jianfeng Feng, Feng Zhang, Li Jin, Zilong Qiu, Xiaohong Gong, Hongyan Wang, was originally published electronically on the publisher’s internet portal on 6 April 2022 without open access. With the author(s)’ decision to opt for Open Choice the copyright of the article changed on 25 April 2022 to © The Authors 2022 and the article is forthwith distributed under a Creative Commons Attribution 4.0 International License, which permits use, sharing, adaptation, distribution and reproduction in any medium or format, as long as you give appropriate credit to the original author(s) and the source, provide a link to the Creative Commons licence, and indicate if changes were made. The images or other third party material in this article are included in the article’s Creative Commons licence, unless indicated otherwise in a credit line to the material. If material is not included in the article’s Creative Commons licence and your intended use is not permitted by statutory regulation or exceeds the permitted use, you will need to obtain permission directly from the copyright holder. To view a copy of this licence, visit http://creativecommons.org/licenses/by/4.0/.

