# Supplementary material for: A recurrent SHANK1 mutation implicated in autism spectrum disorder causes autistic-like core behaviors in mice via downregulation of mGluR1-IP3R1-calcium signaling
Source: Mol Psychiatry. 2022 Apr 6;27(7):2985–98. doi: 10.1038/s41380-022-01539-1 (PMC9205781; doi:10.1038/s41380-022-01539-1)
Supplement: Supplementary file 1 — Supplementary information [file 41380_2022_1539_MOESM1_ESM.docx]

**Supplementary Information**

**A recurrent *SHANK1* mutation implicated in autism spectrum disorder causes autistic-like core behaviors in mice via downregulation of mGluR1-IP3R1-calcium signaling**

Yue Qin, Yasong Du, Liqiang Chen, Yanyan Liu, Wenjing Xu, Ying Liu, Ying Li, Jing Leng, Yalan Wang, Xiao-Yong Zhang, Jianfeng Feng, Feng Zhang, Li Jin, Zilong Qiu, Xiaohong Gong, Hongyan Wang

**Summary**

The supplementary information file includes sections of Supplementary Methods, Supplementary Figures and Supplementary Tables.

**Supplementary Methods**

**Study participants**

A total of 615 patients with ASD (530 males and 85 females) were enrolled from the Department of Child and Adolescent Psychiatry, Shanghai Mental Health Center, Shanghai Jiao Tong University School of Medicine (Shanghai, China), between 2010 and 2013. They came from unrelated families and were diagnosed with ASD according to the criteria of the Diagnostic and Statistical Manual of Mental Disorders, fourth Edition (DSM-IV) of the American Psychiatric Association (APA). The parents of the patients with ASD were evaluated with the Self-Rating Depression Scale (SDS) and Self-Rating Anxiety Scale (SAS). All 503 healthy controls (414 males and 89 females) were recruited from Fudan University (Shanghai, China) from 2010 to 2014. All of the participants were of Han Chinese ethnicity based on the records of the household registration system, which provides demographic information of at least three generations. The average ages (years) of the ASD patients and the controls were 5.81±0.13 and 18.59±0.05 (mean ± standard error (SE)), respectively. This study was approved by the Ethics Committees at Shanghai Jiao Tong University School of Medicine and at Fudan University. Informed consent was obtained from the controls and the guardians or the parents of the children with ASD.

**Mutation screening of the *SHANK1* gene**

Approximately 1 to 3 mL of peripheral blood was collected from each subject in this study. Genomic DNA was extracted using a Mammalian Genomic DNA Extraction Kit (Lifefeng, China). The human gene *SHANK1* (NM_016148.5), mapped to 19q13.33 and consisting of 24 exons, encodes a 2161-amino acid protein (NP_057232.2). For each individual, exons covering the coding sequence (CDS) and adjacent splice sites were amplified by polymerase chain reaction (PCR) for mutation screening. All primers for Sanger sequencing were designed with PRIMER 3 (http://bioinfo.ut.ee/primer3-0.4.0/) and are shown in Supplementary Table 7. For sequencing, the PCR products were pretreated with a mixture of 10 units of Exo I and 1 unit of FastAP (Thermo Fisher Scientific, USA). BigDye Terminator v3.1 and HiDi Formamide (Applied Biosystems, USA) were used for the direct sequencing reaction and subsequent denaturation, respectively. The final products were sequenced on a 3730xl Genetic Analyzer (Applied Biosystems, USA) according to the manufacturer’s instructions. All results were analyzed with Genalys v2.8.3. Identified rare mutations were then confirmed independently by bidirectional resequencing. DNA samples from the parents of those ASD children carrying the mutations were also amplified and sequenced. Paternity testing for specific subjects was performed by the SiFaSTR^TM^ 23-plex system, which is a highly applicable system for the Chinese population and consists of 21 autosomal-STR (A-STR) loci, one Y-Indel and one amelogenin locus, as previously described ([1](#_ENREF_1)).

**Gene filtration and bioinformatics analyses**

Rare (minor allele frequency, MAF <1%) case-specific protein-altering variants were preferred in this study. Missense, nonsense, frameshift and splicing-site variants were also taken into consideration. The 503 healthy control subjects were the primary controls. Variant frequencies based on different ethnic subgroups from the 1000 Genomes Project ([2](#_ENREF_2)), the Exome Aggregation Consortium (ExAC) and the Genome Aggregation Database (gnomAD) ([3](#_ENREF_3), [4](#_ENREF_4)) as well as the Chinese Millionome Database (CMDB) ([5](#_ENREF_5)) were also referenced as controls. Multiple sequence alignment across species was performed with the UniProt server, while evolutionary conservation was scored using PhyloP and PhastCons ([6](#_ENREF_6)). Single-nucleotide variants (SNVs) were evaluated for pathogenic potential with SIFT ([7](#_ENREF_7)), PolyPhen-2 ([8](#_ENREF_8)), MutationTaster2 ([9](#_ENREF_9)), CADD ([10](#_ENREF_10)) and DANN ([11](#_ENREF_11)) tools.

**Plasmid construction, cell culture and transfection**

The construct pGW1-CMV-HA-*Shank1* (rat *Shank1* mRNA, AF131951.1) was a gift from Dr. Sala ([12](#_ENREF_12)). Eight types of point mutations in rat *Shank1* (A612V, R874H, P1806L, D1926N, G2026R, G2037D, A612V+R874H and D1926N+G2037D), which is evolutionarily conserved compared with the human sequence, were made from the wild-type (WT) plasmid by site-directed mutagenesis using a MutanBEST Kit (Takara, Japan). The primers used for plasmid construction are listed in Supplementary Table 8. All constructs were checked by bidirectional sequencing and assessment of the expression of proteins with the expected molecular weights in HEK-293T cells.

Human HEK-293T cells were grown in DMEM supplemented with 10% FBS (Corning, USA) and 1‱ Plasmocin (InvivoGen, USA) at 37°C in 5% CO_2_. About 80% confluent HEK-293T cells were transfected with pGW1-CMV-HA-*Shank1* plasmids carrying WT or mutant *Shank1* by using Lipofectamine 2000 (Invitrogen, USA). Hippocampal neuron cultures were prepared from E18-E19 rats as described previously ([12](#_ENREF_12)). Medium-density neurons (150-200 cells/mm^2^) were plated and grown in Neurobasal Medium containing B-27 supplements, 0.5 mM L-glutamine and 100 U/mL Penicillin-Streptomycin (Gibco, USA) and maintained at 37°C in 5% CO_2_. The neurons were transfected with pGW1-CMV-HA-*Shank1* plasmids carrying WT or mutant *Shank1* by using a PEI-Transferrinfection Kit (Invitrogen, USA). Furthermore, cultured neurons were cotransfected with a green fluorescent protein (GFP) plasmid.

**Immunofluorescence**

After transfection at DIV 8, neurons were fixed in 4% paraformaldehyde (PFA) and 4% sucrose at room temperature (RT) for 10 min at DIV 18. Primary and secondary antibodies were diluted in GDB buffer (30 mM phosphate buffer, pH 7.4, containing 0.2% gelatin, 0.5% Triton X-100, and 0.8 M NaCl). A mouse anti-GFP antibody and a FITC-conjugated secondary antibody as well as a rabbit anti-HA antibody and a Cy3-conjugated secondary antibody (Roche, Switzerland) were used. As previously described ([12](#_ENREF_12)), transfected neurons were selected randomly, and fluorescence images were acquired with an MRC1024 confocal microscope (Bio-Rad, USA) using a 63× oil objective with sequential-acquisition setting at 1024×1024 pixels. Each image was a z-series projection taken at an interval of 0.37 μm. Morphometric analyses were performed by using MetaMorph Image Analysis Software (Universal Imaging, USA). For dendritic spine measurement, the length and head width of each spine present on the dendrites were manually traced and automatically measured, while the spine density was calculated simultaneously.

**Generation and breeding of *Shank1* R882H-knock-in (KI) mice**

The CRISPR/Cas9 gene editing technique was employed to generate *Shank1* R882H-KI mice. Single-guide RNA (sgRNA) was designed (<http://crispr.mit.edu/>) and cloned into the pX330 vector. Cas9 mRNA and sgRNA were transcribed with an mMESSAGE mMACHINE T7 Ultra Kit and a MEGAshortscript T7 Kit (Invitrogen, USA), respectively ([13](#_ENREF_13)). RNAs were purified with a MEGAclear Kit (Invitrogen, USA). A single-stranded oligo-deoxynucleotide (ssODN) donor containing the target mutation R882H was synthesized and purified by PAGE. A mixture of Cas9 mRNA (100 ng/µL), sgRNA (50 ng/µL) and ssODN (100 ng/µL) was microinjected into zygotes isolated from C57BL/6N mice from Beijing Vital River Laboratory. Candidate off-targets were selected by the CRISPR design tool (<http://crispr.mit.edu/>) with high-risk scoring and verified by direct sequencing of genomic DNA of founder mice. The founder mice from CRISPR/Cas9 microinjection were backcrossed to WT mice (C57BL/6N) for three generations to eliminate potential off-target mutations before any experiments. The mouse pups were genotyped by PCR amplification of tail DNA and Sanger sequencing of the PCR products. The sequences used for CRISPR/Cas9 editing and the primers used for genotyping of the mutant site (R882H) are listed in Supplementary Table 8.

Mice were maintained under a 12 h/12 h light/dark cycle at 22-25°C and 40–50% humidity with standard food and water available ad libitum. The offspring of mating pairs were genotyped on postnatal day 14 (P14) and weaned on P21. All three genotypes of animals were derived from intercrosses of heterozygotes, and males were used in all experiments. WT littermates were used as a control for *Shank1* mutants. Invasive operations on animals were performed under anesthesia with pentobarbital sodium (50 mg/kg, i.p.), and all efforts were made to minimize animal suffering. A status of being blind to the genotype was maintained across all procedures during all the assays except biochemical analyses.

This study was approved by the Ethics Committee of Animal Experimentation at Fudan University. All procedures for animal care and animal experiments were carried out in accordance with the guidelines of the Care and Use of Laboratory Animals proposed by Fudan University and Shanghai Municipality, PR China. The protocol was approved by the Science and Technology Commission of Shanghai Municipality (Permit Number: SYXK (hu) 2020-0032).

**Behavioral studies**

A battery of behavioral experiments were performed with age-matched male littermates (8-22 weeks) during the light cycle based on previously published methods ([14-24](#_ENREF_14)) with modifications. The age gap of the mice was less than 3 weeks in each type of behavioral test. The mice were gently handled for at least 3 min/day for 7 days prior to testing. All home cages containing mice were transported to the testing room at least 60 min before the start of testing.

***Three-chamber social interaction and social novelty test***

A rectangular box (61.5×60.5×31 cm^3^) was used, made with a 20.5-cm-wide center chamber and two 20.5-cm-wide side chambers in dim-light conditions (15-16 lux). Doors within the two partitions allowed access to each chamber. Mice used as strangers (8-week-old male C57BL/6N mice) had no prior contact with the subjects and had been habituated to the apparatus for 30 min/day for three consecutive days before all the tests. For both experiments, the subject mice had an adaptation period at the beginning that consisted of exploration for 10 min with the doors closed followed by exploration for 10 min with the doors open.

*Experiment I* Social interaction was measured with a group of mice. The stranger #1 mouse was placed into an inverted wire cup in one side chamber, and the novel object, that is, an empty inverted wire cup, was placed in the other. The subject mouse was allowed to explore for a test session of 10 min. The locations of the stranger #1 and novel object were altered across subjects.

*Experiment II* Experiment II was performed with another group of mice. In phase I (the interaction phase), as in Experiment I, the stranger #1 was positioned into an inverted wire cup in one side chamber, and the subject was allowed to freely move for a test session of 10 min. In phase II (the novelty preference phase), the stranger #2 was placed into a cup in the other side chamber. The subject was placed again in the center for another 10-min test session. The positions of the stranger #1 and #2 were changed between trials.

Each test session was recorded, and the total number of entries into side chambers, the time spent in each side chamber and the time spent sniffing each target were analyzed by EthoVision XT v11.5 (Noldus, USA). We excluded data when an animal had not visited either side chamber in either the adaptation or test session.

***Marble-burying (MB) assay***

Standard polycarbonate rat cages (47×26×31.5 cm^3^) with fitted filter-top covers were used. Preparation involved filling the cages with corncob bedding to a depth of 5 cm and then gently placing glass marbles (1.5 cm) with on the surface precisely in a 4×5 arrangement. The subject mouse was placed at a corner of the cage, far from the marbles, and allowed to stay in the cage undisturbed for 30 min. The number of marbles buried (>50% volume covered by bedding material) was scored for each mouse.

***Open field (OF) test***

The apparatus (40×40×40 cm^3^) was illuminated with overhead lighting at 150 lux. Each subject was placed in the center for a test session of 30 min. The movement time, total distance traveled and distance traveled in the center (1/4 area) were video-recorded and analyzed with EthoVison XT v11.5 (Noldus, USA). The center distance ratio was calculated as the distance the subject traveled in the 1/4-arena center/total distance traveled.

***Light-dark (LD) exploration***

The apparatus was a cage (50×30×30 cm^3^) separated into a light compartment (30 cm, transparent, lit at 400 lux) and a dark compartment (20 cm, black and opaque, 4 lux) by a partition. A door at the bottom of the partition allowed mice to move freely between the two compartments. The subject mouse was placed into the light compartment on a side away from the partition and allowed 10 min of exploration. Data, including the number of transitions between the two compartments and the time spent in the light compartment, were collected and analyzed with EthoVison XT v11.5 (Noldus, USA).

***Elevated plus maze (EPM)***

The elevated plus maze apparatus comprised two open arms (35×6.5×0.5 cm^3^) and two closed arms (35×6.5×19.5 cm^3^) that extended from a center platform of 6.5×6.5×0.5 cm^3^). The apparatus, with a white floor and transparent walls, was elevated 50 cm above the floor, under a light intensity of about 100 lux. Each subject mouse was placed in the center area facing an open arm and allowed to explore for 10 min. Each session was recorded by a ceiling-mounted camera and analyzed with EthoVison XT v11.5 (Noldus, USA). The number of total entries into both the open and closed arms was the measure of general exploratory activity. The time spent in open arms and the entries into open arms served as the index of anxiety-like behavior.

***Novel object recognition (NOR)***

The novel object recognition test was conducted in an open field apparatus (40×40×40 cm^3^) under dim light (15-20 lux). One day before the experiment day, the subject mouse was habituated to the arena for 30 min. In the training stage, the mouse was allowed to explore two identical objects in the box for 10 min. One hour later, the test stage was performed for recognition memory. In this stage, one of the objects was replaced by another novel object, in different shape, and the exploration time in 10 min for one novel object (N) and one familiar object (F) was measured manually. The discrimination index was calculated as (time in exploring N- time in exploring F)/( time in exploring N+ time in exploring F). We excluded data when an animal does not meet the minimum exploration time of 20 s in any stage.

***Barnes maze (BM) assay***

The Barnes maze consisted of a white circular platform (122 cm in diameter and 80 cm in height) containing 40 evenly spaced holes (5 cm) that was lit at 250 lux, with large shapes on the walls as visual cues. An escape box was placed underneath a designated target hole. During adaptation, the mouse was allowed to move to the escape box under gentle guidance and to stay inside for 2 min with a lid. During training, the mice finished 15 trials in 4 consecutive days. For each trial, the mouse was free to search for the escape box for 3 min. Once the mouse entered, the trial was stopped, and the mouse was kept inside for 2 min with a lid. An intertrial interval of 15 min, during which the mice were kept in their home cages in the dark, was used. A probe trial for assessment of short-term or long-term retention was conducted on day 5 or day 12, respectively. The subject was allowed to explore with no escape box for a fixed period of 90 s. The sessions were recorded with a video-tracking system controlled by EthoVison XT v11.5 (Noldus, USA). The parameters measured included the number of total errors and latency before entering the escape box during the training period as well as number of total errors and the percentage of time spent in the target quadrant that previously contained the escape box during the probe period.

**Structural magnetic resonance imaging (sMRI)**

Mice (19-22 weeks) were subjected to a brain magnetic resonance imaging (MRI) study. The animals were immobilized and anesthetized with 5% isoflurane for induction and 1.5-2% for maintenance during all imaging experiments. Respiration was monitored to be stable, and a rectal temperature of 37°C was maintained throughout the experiments using a warm-air feedback system (SA Instruments, USA).

*In vivo* sMRI was performed on an 11.7 T BioSpec 117/16 USR MRI system equipped with a CryoProbe (Bruker BioSpin, Germany). An 89 mm volume coil was used for transmission, and a 4-channel CryoProbe coil was used for receiving. High-resolution T2-weighted (T2W) images of the brain were acquired using a rapid acquisition with relaxation enhancement (RARE) sequence with the following parameters: TE/TR, 40 /4753 ms; RARE factor, 8; spatial resolution, 80×80×400 μm^3^.

Preprocessing of T2W images was performed using Statistical Parametric Mapping 8 (SPM8) for MATLAB 2015b (MathWorks, USA). Intergroup differences in local gray matter volume (GMV) were analyzed with voxel-based morphometry (VBM) ([25](#_ENREF_25)) based on a mouse atlas ([26](#_ENREF_26)). Specifically, the SPMMouse Toolbox was used to perform VBM processing. After registration, structural images were segmented into gray matter (GM), white matter (WM) and cerebrospinal fluid (CSF), and then used to create a DARTEL template. A 0.35 mm FWHM Gaussian kernel was used to smooth the modulated and normalized GM segments. Student’s *t*-test was performed voxel-by-voxel to determine the GMV differences between KI-HOM and WT mice. Significant clusters were identified as >200 voxels with FDR-corrected *P* <0.05.

**Golgi staining**

Dendritic spine histological analysis was performed with an FD Rapid GolgiStain Kit (FD Neurotechnologies, USA) following the manufacturer's protocol as previously described ([27](#_ENREF_27), [28](#_ENREF_28)). Briefly, mouse (5-6 weeks) brains were dissected to obtain tissue blocks containing the hippocampi ([29](#_ENREF_29)). The brain blocks were rinsed with PBS, placed in impregnation solution containing mercuric chloride, potassium dichromate and potassium chromate (Solutions A and B), and stored in the dark at RT for 14 days. The brains were transferred into a solution containing sucrose (Solution C), incubated at 4°C for 5-7 days until the residual water was driven from the tissues, rapidly frozen in liquid nitrogen and stored at −80°C. Next, a cryostat at −22℃ to −20℃ (CM1950, Leica, Germany) was used to obtain 100 μm thick coronal slices. The slices were mounted on 1% gelatin-coated glass slides. After sufficient natural drying, the slices were rinsed and incubated for 10 min in a solution containing silver nitrate (Solutions D and E). The slides were then dehydrated and cleared in baths of graded ethanol and xylene, respectively, cover-slipped with Neutral Balsam Mounting Medium (Sangon Biotech, China), and subsequently stored in the dark. Brightfield microscopy images of pyramidal neurons from the CA1 region were imaged on a laser confocal scanning microscope (LSM 700, Zeiss, Germany) using a 63× oil-immersion objective. Each image was a z-series projection of 30-80 pictures taken at depth intervals of 0.46-0.5 μm. For measurements, neurons that satisfied the following criteria were selected for data collection: (1) the cell type was identifiable, (2) the cell body and dendrites were completely impregnated, (3) the stained neuron was isolated from neighboring neurons, and (4) the dendrites were not truncated. Morphometric reconstruction and calculation were performed using ImageJ (NIH, USA) and Neurolucida v9.0 (MBF Bioscience, USA). Dendritic spine density, spine length and spine head width were measured 10-15 μm away from the beginning of the secondary apical dendrites of pyramidal neurons. The spine density was defined as the average number of spines per 10 microns of dendritic length.

**Transmission electron microscopy (TEM)**

This experiment was carried out according to previously published protocols ([30](#_ENREF_30), [31](#_ENREF_31)) with modifications. Mice (5-6 weeks) were deeply anesthetized and perfused with PBS (pH 7.4) followed by ice-cold 4% PFA in phosphate buffer (PB; in M: 0.02 NaH_2_PO_4_, 0.08 Na_2_HPO_4_; pH 7.4). The hippocampal tissues were dissected, cut into 1 mm^3^ cubes and fixed overnight with 2% glutaraldehyde and 2% PFA in PB at 4°C. Coronal sections (50 µm) were cut on a vibratome (VT1200S, Leica, Germany) and postfixed in 1% OsO_4_ for 1 h. After dehydration in ethanol (50%, 70%, 80%, 90%, 95%, 100%) and acetone (100%) for 20 min each and infiltration with graded Epon/Spurr resin, the material was finally impregnated with 100% Epon/Spurr resin and embedded in molds. Ultrathin (70 nm) sections were cut on an ultramicrotome (EM UC7, Leica, Germany), mounted on 200-mesh Metaxaform Copper Rhodium grids and poststained with 2% uranyl acetate for 15 min and Sato’s lead citrate for 7 min. Electron micrographs were randomly acquired of the CA1 region at 10000× or 15000× magnification with a transmission electron microscope (HT7800, Hitachi, Japan) at 120 kV accelerating voltage. Asymmetric synapses with clearly visible synaptic structures were considered ([32](#_ENREF_32), [33](#_ENREF_33)). Postsynaptic densities (PSDs) can be divided into two groups: macular PSDs which have continuous sheets, and perforated PSDs which are discontinuous ([34](#_ENREF_34)). PSD measurements were performed with ImageJ independently by two investigators. The length was measured directly, while the average thickness was calculated as the average area/length. In addition, the entire length of all fragments was measured as a single value for perforated PSDs.

**Preparation of hippocampal slices and** **electrophysiology**

The procedures were conducted as previously described ([32](#_ENREF_32)) with modifications. The cutting solution (CS; in mM: 10 MgSO_4_, 2.5 KCl, 30 NaHCO_3_, 1.2 NaH_2_PO_4_, 0.5 CaCl_2_, 30 glucose, 92 NMDG, 20 HEPES, 5 Na-ascorbate, 3 Na-pyruvate, 2 thiourea and 64.26 HCl; pH 7.4) and artificial cerebrospinal fluid (ACSF; in mM: 119 NaCl, 1.3 MgSO_4_, 2.3 KCl, 26.2 NaHCO_3_, 1 NaH_2_PO_4_, 2.5 CaCl_2_ and 12 glucose; pH 7.4) were prepared, pre-equilibrated with 95% O_2_ and 5% CO_2_ for at least 0.5 h and stored at 4°C and 32°C, respectively. In the following steps, CS and ACSF were always gassed with 95% O_2_ and 5% CO_2_. Mice (5-6 weeks) were perfused with CS after anesthesia. Brain tissues containing the hippocampus were cut into coronal slices of 350 µm on the vibratome in CS at 4°C, transferred into ACSF and incubated for recovery at 32°C for 0.5 h followed by RT (23-25°C) for 1-2 h prior to recordings.

For extracellular recordings, the stimulation electrode, a bipolar tungsten electrode (WPI, USA), was placed in Schaffer collateral fibers in CA3, and a recording electrode filled with ACSF, pulled from borosilicate glass capillaries (1.5 mm OD; Sutter Instrument, USA) with a micropipette electrode puller (Puller-97, Sutter Instrument, USA), was placed into the stratum radiatum in CA1. Field excitatory postsynaptic potentials (fEPSPs) were recorded in stratum radiatum, evoked by stimulation from Schaffer collateral fibers (100-μs pulses every 30 s) with Master-8 Pulse Stimulator (A.M.P.I., Israel). Once the stimulation intensity was adjusted to give fEPSP slopes that were 40-50% of the maximal response, a stable baseline was recorded under this stimulation strength for 10 min at an interval of 30 s. After that, long-term potential (LTP) was induced with theta burst stimulation (TBS; 4 bursts of 4 pulses at 100 Hz spaced at 200 ms intervals, repeated 4 times at 10 s intervals), and the fEPSP was recorded for 60 min. LTP induction was considered successful when a significant increment over 20% of the baseline and a long duration of over 60 min were seen in this recording.

For miniature excitatory postsynaptic current (mEPSC) measurements, pyramidal neurons in CA1 were patched with recording electrodes made from glass capillaries at −70 mV in voltage-clamp mode. The mEPSC events were recorded with an internal solution (in mM: 115 CH_3_CsO_3_S, 20 CsCl, 10 HEPES, 2.5 MgCl_2_, 4 Na_2_ATP, 0.4 Na_3_GTP, 10 Na-phosphocreatine and 0.6 EGTA; pH 7.2; 300 mOsm/L) in the presence of 1 μM tetrodotoxin and 100 μM picrotoxin (Sigma-Aldrich, Germany). Signals were collected for 5 min in gap-free mode, which were considered valid data when the range of series resistance was smaller than 20% during the recording.

Extracellular recordings were performed using MultiClamp, digitized with a Digidata 1440A device and analyzed with pClamp v10.6. Whole-cell mEPSCs were recorded via an Axopatch 700B amplifier equipped with a Digidata 1440A digitizer and analyzed with MiniAnalysis v10.6.

**RNA-Seq analysis for transcriptomics**

Total RNA (10 µg) was extracted from mouse (5-6 weeks) hippocampus homogenates using TRIzol reagent (Life Technologies, USA), and the RNA integrity number (RIN) was assessed with a 2100 Bioanalyzer system (Agilent Technologies, USA). Qualified total RNA was purified with an RNAClean XP Kit (Beckman Coulter, Germany) and an RNase-Free DNase Set (Qiagen, Germany). A library based on poly-A-tailed mRNA selection was prepared according to Illumina standard instructions with multiple kits. The purified libraries were quantified with a Qubit 2.0 Fluorometer (Invitrogen, USA) and validated with a 2100 Bioanalyzer system. Clusters were generated by cBot with the library diluted to 10 pM and then sequenced on an Illumina HiSeq 2500 system (Illumina, USA). High-quality reads were mapped to the mouse reference genome mm10 in the ENSEMBL database for sequence analysis. The mRNA abundance was normalized as the FPKM values transformed from reads. The RNAs of differentially expressed genes (DEGs) were identified as those with a fold change greater than 2 and an FDR-corrected *P* value less than 0.05 for three R882H-KI HOM mice compared with three WT littermates. Enrichment analysis of the DEGs in KEGG pathways was conducted with DAVID Bioinformatics Resources (version 6.8; <https://david.ncifcrf.gov/>) ([35](#_ENREF_35), [36](#_ENREF_36)), and a significance level of 0.05 after Bonferroni correction was used.

**Preparation of postsynaptic density (PSD) fractions**

PSD fractions were extracted from mouse (5-6 weeks) whole brains or brain subregions, including the frontal cortex, hippocampus and cerebellar cortex, using previously described protocols ([37](#_ENREF_37), [38](#_ENREF_38)) with some modifications. All procedures were performed at 4°C. All buffers were supplemented with protease (Roche, Switzerland) and phosphatase inhibitors (APExBIO, USA). Tissue from mouse brains was homogenized in HEPES-buffered sucrose (0.32 M sucrose, 4 mM HEPES, pH 7.4) and centrifuged at 1000 x *g* for 10 min. The supernatant (S1) was transferred to a new tube and centrifuged at 12000 x *g* for 20 min to obtain the crude synaptosomal pellet (P2). This fraction, from 3-4 mouse brains, was further fractionated by sucrose density gradient centrifugation (0.8/1.0/1.2 M HEPES-buffered sucrose solution) at 150000 x *g* for 2 h after lysing using ddH_2_O and 4 mM HEPES. The synaptic plasma membrane (SPM) was collected at the bottom of the 1.0/1.2 M interphase. To obtain the so-called crude PSD fraction, the SPM pellet was resuspended in a solution of 0.54% Triton X-100 in 50 mM HEPES/2 mM EDTA, rotated for 15 min and centrifuged at 35000 x *g* for 30 min. Finally, the PSD fractions were prepared for further quantitative LC-MS/MS and immunoblot analyses.

**Label-free quantitative proteomics**

Label-free LC-MS/MS was carried out for quantitative proteomic analysis of crude PSDs derived from the mouse hippocampus as previously described with minor modifications ([39-41](#_ENREF_39)). Prior to tryptic digestion, PSD fractions were lysed in SDT buffer (2% SDS, 100 mM DTT, 100 mM Tris-HCl, pH 7.6). After quantification with a BCA Protein Assay Kit (Beyotime, China), the proteins were digested according to a filter-aided sample preparation (FASP) procedure ([42](#_ENREF_42)). Tryptic peptides were analyzed for samples from three pairs of R882H-KI HOM versus WT mice using a Q Exactive mass spectrometer (Thermo Fisher Scientific, USA); each sample was run for 2 h. The raw data were processed with MaxQuant software and searched against the UniProt mouse database, with the FDR cutoff set to 0.01 at both the peptide and protein levels. The label-free quantification (LFQ) intensity of each protein was calculated by MaxQuant. Only proteins identified by at least two peptides with a minimum length of seven amino acids were considered. Additionally, to be included in the final dataset, a protein had to be identified in at least two biological replicates in at least one condition (in either R882H-KI HOM or WT hippocampi). Mitochondrial proteins were also excluded due to their inevitable contamination during PSD preparation ([37](#_ENREF_37), [43](#_ENREF_43)). Finally, quantitative analysis was performed based on the LFQ intensity for protein abundance alterations between R882H-KI HOM and WT hippocampi. To be included in the list of regulated proteins, proteins had to be statistically significant at 0.01 (Student’s *t*-test) and display a log_2_ ratio of at least ±0.58 (a fold change greater than 1.5) between mutant and WT mice, or they had to meet the criterion of identification only in mutant or WT samples. Pathway enrichment based on the KEGG database for the list of regulated proteins was carried out using DAVID Bioinformatics Resources with a significance threshold of 0.01 after Bonferroni correction.

**Immunoblot analysis**

HEK-293T cells were harvested and lysed in ice-cold RIPA buffer (Beyotime, China) with protease inhibitors for 10 min after transfection for 48 h. PSD fractions from mouse brains were lysed for 15 min in a 4:1 mixture of 0.54% Triton X-100/0.5% SDS in 50 mM HEPES/2 mM EDTA solution on ice containing protease and phosphatase inhibitors. Both cell and PSD lysates were quantified with a BCA assay. Western blotting was performed using standard protocols on PVDF membranes. Multiple primary antibodies were used. To detect transfected WT or mutant HA-tagged SHANK1 in 293T cells, antibodies against HA (M20003) or GAPDH (G8795, Sigma-Aldrich, Germany) were used. For detecting PSD proteins, the following antibodies were purchased from commercial suppliers: antibodies against Shank1 (TA309857, OriGene, USA), β3-Tubulin (ab78078), GluN1 (ab109182) (both from Abcam, UK), GluA1 (AF2473, Beyotime, China), ERK1/2 (4695), phospho-ERK1/2 (Thr 202, Tyr 204) (4370), mGluR1 (12551) (all from Cell Signaling, USA), Homer1 (12433-1-AP), Homer2 (11143-1-AP), Homer3 (16624-1-AP), Ppp3ca (13422-1-AP), Ppp3cb (55148-1-AP), IP3R1 (19962-1-AP), PSD-95 (20665-1-AP), GluA2 (11994-1-AP), GluA4 (23350-1-AP) and GluN2A (19953-1-AP) (all from Proteintech, USA). Multicolor broad or high-range protein ladders (Thermo Fisher Scientific, USA) were used for each western blotting. Detection was performed using ECL reagent on a Tanon 5200 Chemiluminescent Imaging System (Tanon, China). Quantitative analysis of the grayscale value of each band was carried out with ImageJ, and the relative expression levels of specific proteins were normalized to the grayscale value of GAPDH for HEK-293T cell proteins or β3-Tubulin for PSD proteins.

**Measurement of intracellular calcium concentrations**

Hippocampal samples were separated from mice (5-6 weeks) for intracellular calcium concentration detection using a protocol modified from one previously described ([44](#_ENREF_44)). Hippocampal tissues were digested with 0.25% trypsin (Gibco, USA) and DNase I at 50 U/mL (Beyotime, China) for 30 min in a 37°C water bath. Digestion was terminated with DMEM (Gibco, USA) containing 10% FBS (Corning, USA), and then the single-cell suspension was collected, which was washed three times with D-Hanks (Sangon Biotech, China) by centrifugation at 300 x *g* for 8 min. The cells were resuspended in D-Hanks and adjusted to a density of 10^6^ cells/mL after more than 90% cell viability was determined by trypan blue staining (Beyotime, China). The labeled calcium indicator Fluo-4 AM was added to the suspension at 2 μM, accompanied by 0.01% Pluronic F-127 (Beyotime, China). The cell suspension was incubated for 45 min at 37°C in the dark, and the cells were rinsed three times with D-Hanks. After another incubation for 30 min at RT in D-Hanks, the intracellular calcium concentration was measured by flow cytometry on a FACSCalibur (BD Biosciences, USA) at an emission wavelength of 525 nm. The stimuli applied were L-glutamate (G8010, Solarbio, China) and 3,5-dihydroxyphenylglycine (DHPG) (ab120020, Abcam, UK), a specific mGluR1/5 receptor agonist ([45](#_ENREF_45), [46](#_ENREF_46)). The data were analyzed with FlowJo v10.

**Statistical analyses**

Statistical analyses were performed using GraphPad Prism v8.0.2 (GraphPad Software, USA) or R (in RStudio), which were also used to generate graphical illustrations. For immunoblotting and calcium measurement, to correct for the experiment-to-experiment signal intensity variation, each value obtained in an experiment was normalized by the average obtained from all samples in that experiment ([47](#_ENREF_47)). For comparisons between two groups, unpaired Student’s *t*-test was applied to analyze differences in the length and thickness of PSDs, the frequency and amplitude of mEPSCs, and the results of immunoblot analysis. Paired Student’s *t*-test was used for analysis of differences in the time spent in the chamber or spent sniffing the target in the three-chamber test ([48](#_ENREF_48), [49](#_ENREF_49)). The cumulative frequency distributions of PSD or spine lengths and widths were compared using the Kolmogorov-Smirnov test or Kruskal-Wallis test followed by the Kolmogorov-Smirnov test. Comparisons of the percentages of spines with length or head width ≤ 1 µm were conducted by the chi-square test combined with Bonferroni correction. The spine length and head width values in rat neurons and results from the following assays were analyzed with one-way ANOVA: Golgi staining, marble-burying, spatial memory evaluation in Barnes maze, open field test, light-dark test, elevated plus maze test, novel object recognition test, records of body weight and measurements of total frequency or transitions, for which Dunnett’s multiple comparisons test was used for comparisons between the experimental groups and the wild-type control group; and the abundance of Homer family proteins in three brain regions, for which Tukey’s multiple post hoc analysis test was chosen. Data from the LTP recording and the learning procedure in the Barnes maze were analyzed by repeated measures ANOVA followed by the original FDR method of Benjamini and Hochberg and post hoc analysis with Dunnett’s test, respectively. Differences in intracellular calcium concentrations were analyzed by two-way ANOVA followed by Dunnett's multiple comparisons test between two groups. The data are presented as the mean ± SE. All statistical assessments were two-sided, and the corresponding *P* values were calculated, with a signiﬁcance level of 0.05.

**Supplementary Figures**


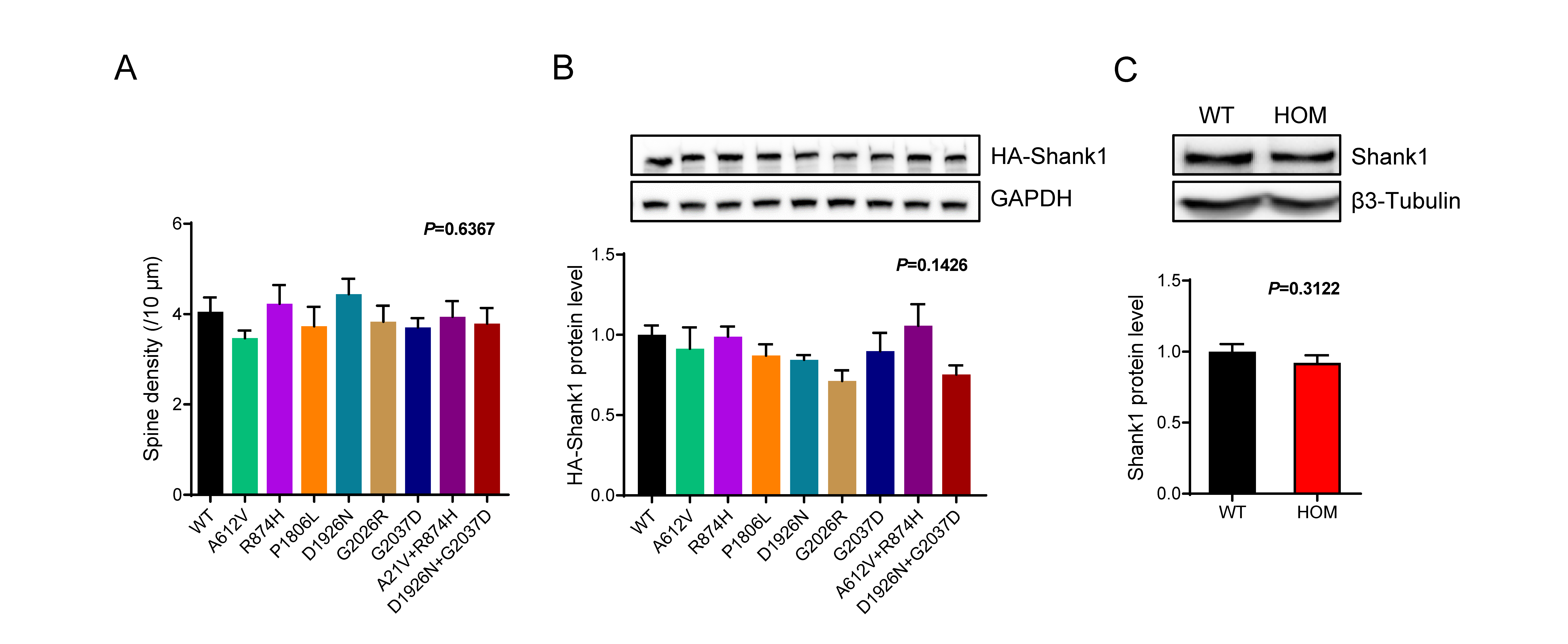


**Supplementary Fig. 1 A** The spine density in cultured neurons derived from the rat hippocampus was not affected by mutant *Shank1* constructs compared with the WT construct. **B** The protein levels of Shank1 were not changed by the mutations, as shown by immunoblot analyses of proteins extracted from HEK-293T cells transfected with WT or mutant *Shank1* constructs (one-way ANOVA), with GAPDH as the loading control. All data shown are the mean ± SE from five independent experiments. **C** The protein levels of Shank1 in PSDs extracted from the whole brains of WT or R882H-KI HOM mice (8 weeks) were quantified by immunoblot analyses. No difference was found between groups (unpaired Student’s *t*-test). All data shown are shown as the mean ± SE of three independent experiments.


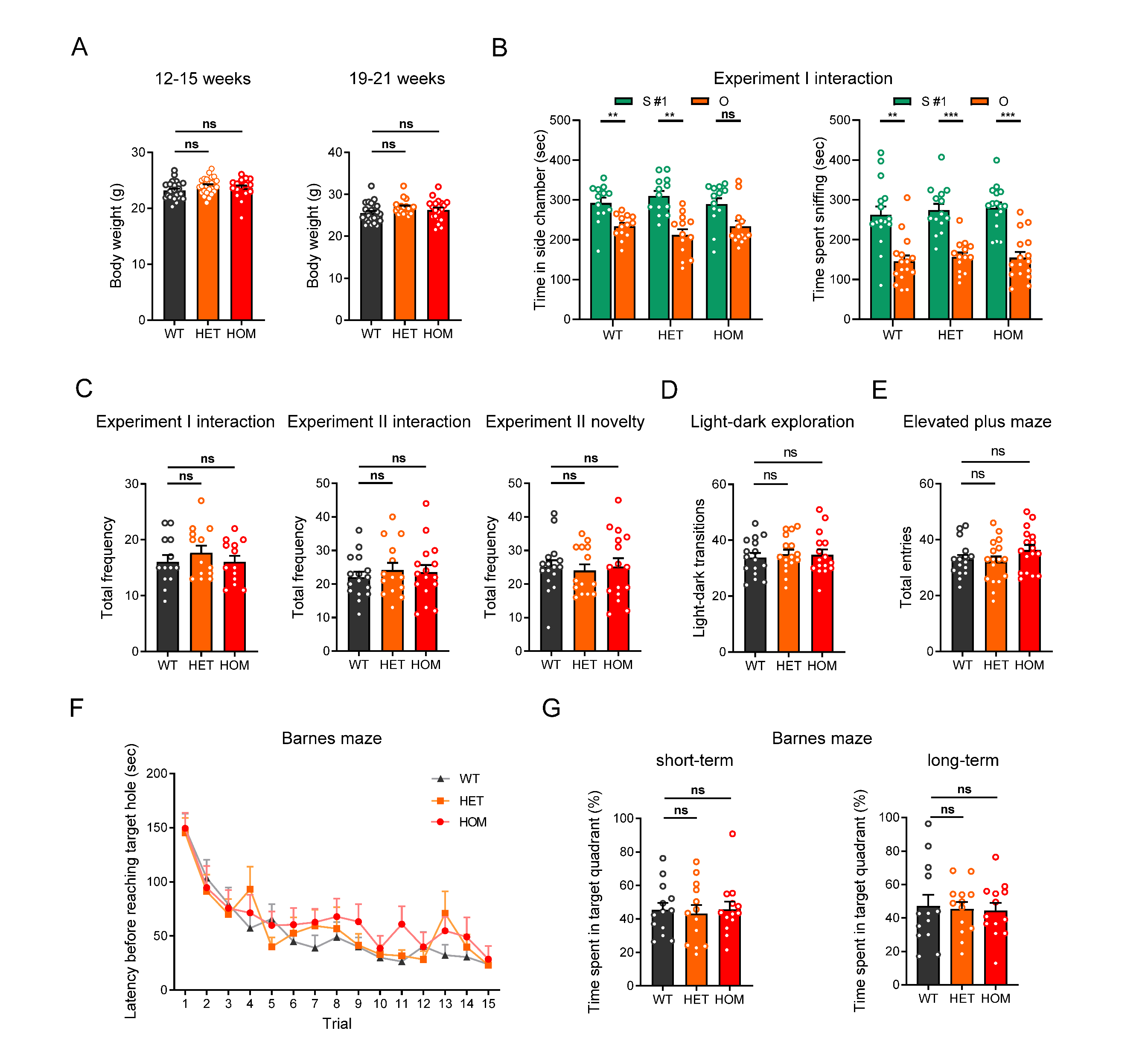


**Supplementary Fig. 2 A** The body weights of animals of three genotypes were measured. No significant differences were obtained. The left panel shows body weights measured at 12-15 weeks (n=16-26 for each genotype). The right panel shows body weights measured at 19-21 weeks (n=18-23 for each genotype). **B** Time in side chamber and time spent sniffing during social interaction measured in Experiments I (n=13 for each genotype). S #1, a stranger mouse placed in one chamber. O, a novel object (inverted wire cup) placed in another chamber. **C** No differences were found in the numbers of entries in the three-chamber social interaction and novelty preference tests in Experiments I and II. **D** The number of transitions between two compartments was comparable among the three genotypes in the light-dark exploration test (n=16 for each genotype). **E** No differences in total entries in the elevated plus maze test (n=16-17 for each genotype). **F** Normal performance of spatial learning was observed in the Barnes maze (n=13 for each genotype), as measured by the latency before reaching the target hole (genotype, *P*=0.4811, trial, *P*<0.0001, interaction, *P*=0.8882). **G** No deficits in short-term and long-term spatial memory were observed in R882H-KI mice, as indicated by the time spent in the target quadrant zone. One-way ANOVA for **A, C-E, G**. Paired Student’s *t*-test for **B**. Repeated measures ANOVA for **F**. All data are presented as the mean ± SE. ns, no significance, **P*<0.05 for the groups.


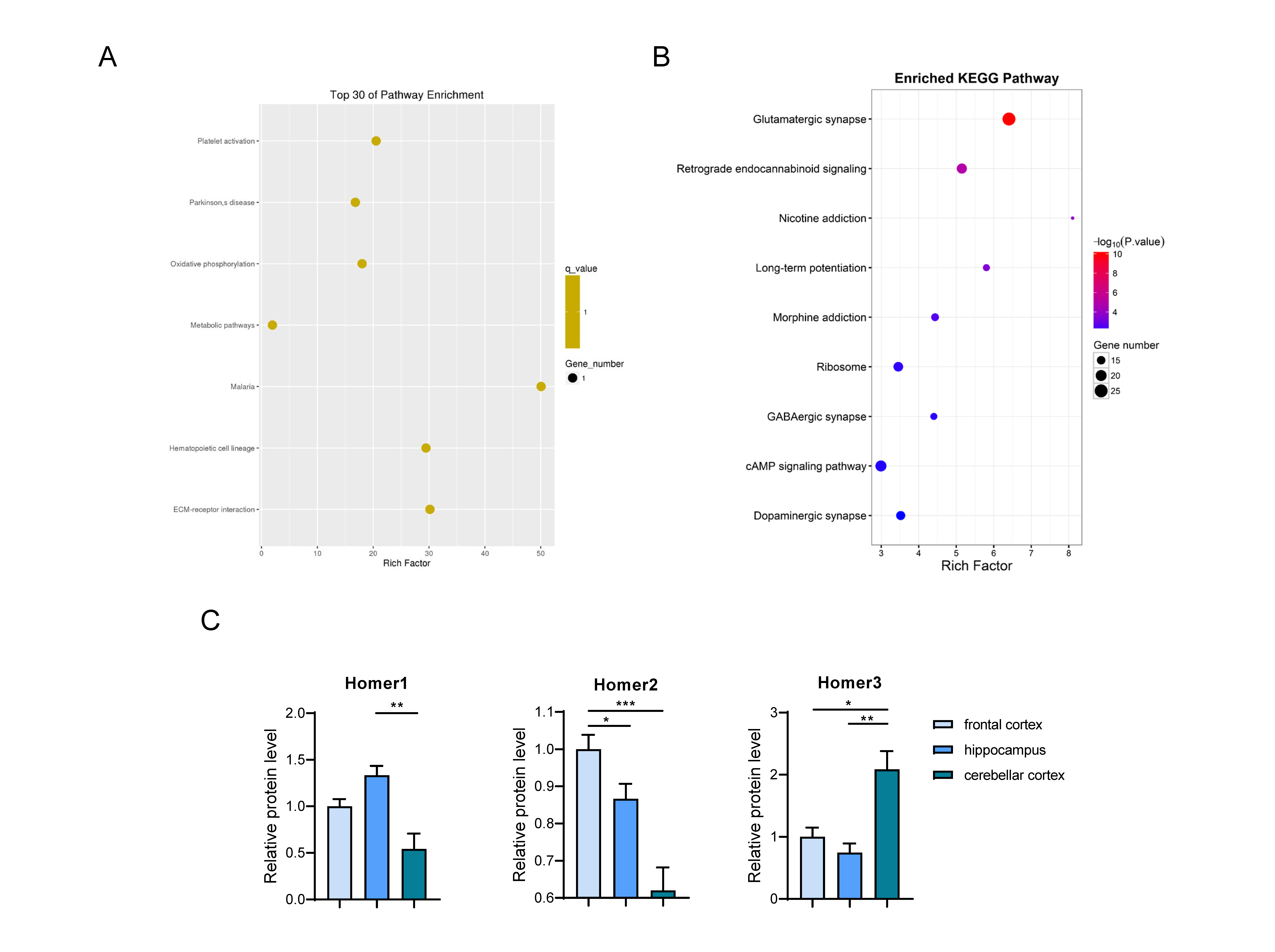


**Supplementary Fig. 3 A** KEGG pathway analysis of the RNA-Seq data showed that no pathway was significantly enriched. The q-value is the *P* value corrected with Bonferroni’s test. A significance level of 0.05 was used. **B** KEGG pathway enrichment of the LC-MS/MS data showed that nine pathways were significantly enriched for a Bonferroni-corrected *P* value significance threshold of 0.01. The top pathway was the glutamatergic pathway, which was investigated further. **C** Protein levels of Homer1/2/3 in PSD fractions from three brain regions. Homer1 was highly expressed in the hippocampus (HP), Homer2 was highly expressed in the frontal cortex (FC), and Homer3 was highly expressed in the cerebellar cortex (CBC) in WT mice. Statistical analysis was performed on data from two or three independent experiments. All data are shown as the mean ± SE. One-way ANOVA followed by Tukey’s multiple comparisons test was used for analyses. **P*<0.05, ***P*<0.01, ****P*<0.001, *****P*<0.0001 for the groups.


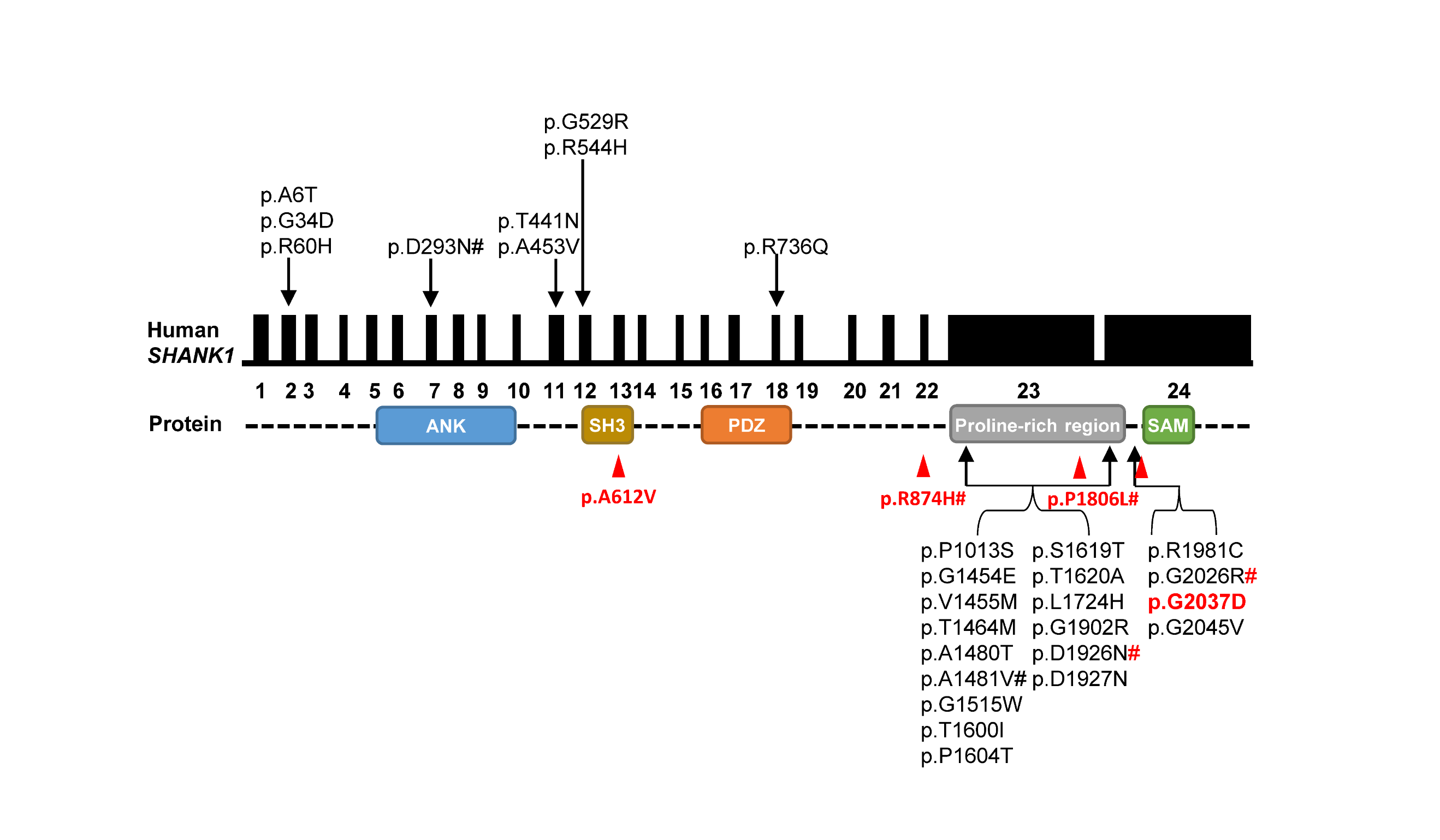


**Supplementary Fig. 4** Missense mutations in patients with ASD from published studies ([50-54](#_ENREF_50)) and from this study. The mutations found in this work are in red. # represents recurrent mutations.

**Supplementary Tables**

**Supplementary Table 1. Overview of *SHANK1* rare variants previously identified in ASD**

| Detected variants | | Variant type | Inheritance pattern | Occurrence | | Study |
| --- | --- | --- | --- | --- | --- | --- |
| DNA change | Amino acid change |  |  | Cases | Controls |  |
| - | - | copy_number_loss  (involving SHANK1 and SYT3) | De novo | 1 | 0 | Sato *et al.*, 2012 ([50](#_ENREF_50)) |
| - | - | copy_number_loss  (involving SHANK1 and CLEC11A) | Familial | 4 | 0 | Sato *et al.*, 2012 |
| c.16G>A | p.A6T | missense_variant | Unknown | 1 | 0 | Leblond *et al.*, 2014 ([51](#_ENREF_51)) |
| c.101G>A | p.G34D | missense_variant | Familial | 1 | 0 | Sato *et al.*, 2012; Leblond *et al.*, 2014 |
| c.179G>A | p.R60H | missense_variant | Familial | 1 | 0 | Sato *et al.*, 2012; Leblond *et al.*, 2014 |
| c.877G>A | p.D293N | missense_variant | Familial | 2 | 0 | Sato *et al.*, 2012 |
| c.1322C>A | p.T441N | missense_variant | Familial | 1 | 0 | Sato *et al.*, 2012; Leblond *et al.*, 2014 |
| c.1358C>T | p.A453V | missense_variant | Unknown | 1 | 0 | Leblond *et al.*, 2014 |
| c.1585G>A | p.G529R | missense_variant | Familial | 1 | 0 | Sato *et al.*, 2012; Leblond *et al.*, 2014 |
| c.1631G>A | p.R544H | missense_variant | Unknown | 1 | 0 | Leblond *et al.*, 2014 |
| c.2207G>A | p.R736Q | missense_variant | Familial | 1 | 0 | Sato *et al.*, 2012; Leblond *et al.*, 2014 |
| c.2458+1G>A | - | splice_site_variant | De novo | 1 | 0 | Wang *et al.*, 2016 ([53](#_ENREF_53)) |
| c.3037C>T | p.P1013S | missense_variant | Familial | 1 | 0 | Sato *et al.*, 2012; Leblond *et al.*, 2014 |
| c.3814_3815insGACGGCGGG | p.L1272delinsRRRV | inframe_indel | Unknown | 1 | 0 | Monies *et al.*, 2019 ([54](#_ENREF_54)) |
| c.4361G>A | p.G1454E | missense_variant | Familial | 1 | 0 | Sato *et al.*, 2012; Leblond *et al.*, 2014 |
| c.4363G>A | p.V1455M | missense_variant | Familial | 1 | 0 | Sato *et al.*, 2012; Leblond *et al.*, 2014 |
| c.4391C>T | p.T1464M | missense_variant | Unknown | 1 | 0 | Leblond *et al.*, 2014 |
| c.4438G>A | p.A1480T | missense_variant | Unknown | 1 | 0 | Sato *et al.*, 2012; Leblond *et al.*, 2014 |
| c.4442C>T | p.A1481V | missense_variant | Familial (1 case), Unknown (1 cases) | 2 | 0 | Sato *et al.*, 2012; Leblond *et al.*, 2014 |
| c.4543G>T | p.G1515W | missense_variant | Familial | 1 | 0 | Sato *et al.*, 2012; Leblond *et al.*, 2014 |
| c.4799C>T | p.T1600I | missense_variant | Familial | 1 | 0 | Sato *et al.*, 2012; Leblond *et al.*, 2014 |
| c.4810C>A | p.P1604T | missense_variant | Unknown | 1 | 0 | Sato *et al.*, 2012; Leblond *et al.*, 2014 |
| c.4855T>A | p.S1619T | missense_variant | Familial | 1 | 0 | Sato *et al.*, 2012; Leblond *et al.*, 2014 |
| c.4858A>G | p.T1620A | missense_variant | Familial | 1 | 0 | Sato *et al.*, 2012; Leblond *et al.*, 2014 |
| c.5171T>A | p.L1724H | missense_variant | Familial | 1 | 0 | Sato *et al.*, 2012; Leblond *et al.*, 2014 |
| c.5704G>A | p.G1902R | missense_variant | De novo | 1 | 0 | Krumn *et al.*, 2015 ([52](#_ENREF_52)); Wang *et al.*, 2016 |
| c.5776G>A | p.D1926N | missense_variant | Familial | 2 | 0 | Sato *et al.*, 2012; Leblond *et al.*, 2014 |
| c.5779G>A | p.D1927N | missense_variant | Unknown | 1 | 0 | Krumn *et al.*, 2015; Wang *et al.*, 2016 |
| c.5941C>T | p.R1981C | missense_variant | Familial | 1 | 0 | Krumn *et al.*, 2015; Wang *et al.*, 2016 |
| c.6076G>A | p.G2026R | missense_variant | Unknown | 1 | 0 | Leblond *et al.*, 2014 |
| c.6134G>T | p.G2045V | missense_variant | Unknown | 1 | 0 | Sato *et al.*, 2012; Leblond *et al.*, 2014 |

**Supplementary Table 2. Psychiatric conditions in parents of patients with ASD**

| **Patient with ASD** | **Sex** | **Mutation** | **Inheritance** | **Psychiatric conditions of father^a^** | **Psychiatric conditions of mother^a^** |
| --- | --- | --- | --- | --- | --- |
| ASD170P | F | c.5417C>T (p.P1806L) | Maternal | N | N |
| ASD184P | M | c.1835C>T (p.A612V)  c.2621G>A (p.R874H) | Maternal | Mild depression | Moderate depression and mild anxiety |
| ASD200P | M | c.5776G>A (p.D1926N)  c.6110G>A (p.G2037D) | Parental  Maternal | Mild depression and mild anxiety | N |
| ASD413P | M | c.6076G>A (p.G2026R) | Maternal | NA | NA |
| ASD488P | M | c.5417C>T (p.P1806L) | *De novo* | Mild depression | N |
| ASD837P | M | c.2621G>A (p.R874H) | *De novo* | Mild depression | Mild depression |

F, female. M, male. N, normal when assessed with the SDS or SAS. NA, no available information.

^a^The parents of the patients with ASD were evaluated with the Self-Rating Depression Scale (SDS) and Self-Rating Anxiety Scale (SAS).

**Supplementary Table 3. General information of genotyping data of 21 A-STR loci, one Y-Indel and one amelogenin locus for the ASD488P and ASD837P trios**

| **STR** | **ASD488P** | **ASD488F** | **ASD488M** | **ASD837P** | **ASD837F** | **ASD837M** |
| --- | --- | --- | --- | --- | --- | --- |
| D3S1358 | 15, 16 | 15, 16 | 15, 16 | 15, 16 | 15, 16 | 16, 16 |
| D5S818 | 11, 13 | 11, 13 | 11,11 | 10, 11 | 11, 12 | 10, 11 |
| D2S1338 | 19, 19 | 19, 24 | 19, 21 | 19, 22 | 19, 22 | 22, 22 |
| TPOX | 8, 12 | 9, 12 | 8, 8 | 9, 11 | 9, 11 | 9, 11 |
| CSF1PO | 12, 14 | 11, 14 | 11, 12 | 10,10 | 10, 11 | 10, 11 |
| Penta D | 11, 13 | 9, 13 | 11, 12 | 9, 12 | 9, 9 | 11, 12 |
| Y-indel | 1 | 1 | - | 1 | 1 | - |
| Amelogenin | X, Y | X, Y | X, X | X, Y | X, Y | X, X |
| TH01 | 9, 9 | 7, 9 | 7, 9 | 7, 9 | 6, 9 | 7, 9 |
| vWA | 14, 14 | 14, 17 | 14, 18 | 14, 17 | 14, 14 | 17, 18 |
| D7S820 | 8, 11 | 11, 12 | 8, 12 | 9, 12 | 9, 11 | 11, 12 |
| D21S11 | 31, 32.2 | 32.2, 32.2 | 31, 31.2 | 32.2, 33.2 | 30, 32.2 | 29, 33.2 |
| Penta E | 11, 11 | 10, 11 | 11, 13 | 15, 16 | 12, 16 | 12, 15 |
| D10S1248 | 13, 15 | 13, 13 | 12, 15 | 13, 13 | 13, 13 | 13, 13 |
| D8S1179 | 12, 12 | 12, 15 | 12, 14 | 13, 18 | 13, 15 | 13, 18 |
| D1S1656 | 14, 17.3 | 14, 17.3 | 15, 17.3 | 13, 17.3 | 13, 16 | 11, 17.3 |
| D18S51 | 14, 14 | 13, 14 | 14, 14 | 11, 13 | 13, 21 | 11, 15 |
| D12S391 | 18, 19 | 18, 22 | 19, 23 | 18, 22 | 18, 22 | 22, 23 |
| D6S1043 | 12, 17 | 12, 18 | 12, 17 | 12, 13 | 11, 12 | 13, 20 |
| D19S433 | 13, 14 | 13, 14 | 13, 13 | 13, 13.2 | 13, 13 | 13, 13.2 |
| D16S539 | 9, 12 | 10, 12 | 9, 9 | 9, 11 | 11, 14 | 9, 12 |
| D13S317 | 11, 11 | 11, 12 | 11, 11 | 10, 11 | 8, 11 | 10, 12 |
| FGA | 23, 25 | 20, 25 | 23, 25 | 18, 23 | 23, 23 | 18, 23 |

P, patient. F, father. M, mother.

In the patient, numbers in blue represent the locus inherited from his father, numbers in red represent the locus inherited from his mother, and numbers in green represent the locus inherited from his father and/or mother.

**Supplementary Table 4. Genes clustered in the glutamatergic synapse pathway and 14 genes identified in the PSD**

| **ID** | **Gene Name** | **Gene Description** | **Location** | **log_2_FC** | ***P* value** |
| --- | --- | --- | --- | --- | --- |
| Q99MK8 | GRK | G protein-coupled receptor kinase 2(Grk2) | pre | —— | - |
| P63328 | **PP2B** | protein phosphatase 3, catalytic subunit, alpha isoform(Ppp3ca) | post | 0.602057 | 0.002304841 |
| P48453 | **PP2B** | protein phosphatase 3, catalytic subunit, beta isoform(Ppp3cb) | post | 0.46625 | 0.000382658 |
| Q9Z2Y3 | **HOMER1** | homer scaffolding protein 1(Homer1) | post | 0.549946 | 0.000579248 |
| Q9QWW1 | **HOMER2** | homer scaffolding protein 2(Homer2) | post | 0.500337 | 0.00778644 |
| Q99JP6 | **HOMER3** | homer scaffolding protein 3(Homer3) | post | —— |  |
| P11881 | **IP3R** | inositol 1,4,5-trisphosphate receptor 1(Itpr1) | post | —— | - |
| Q63844 | **ERK1** | mitogen-activated protein kinase 3(Mapk3) | post | —— | - |
| B2RSH2 | Gi/o | guanine nucleotide binding protein (G protein), alpha inhibiting 1(Gnai1) | both | —— | - |
| P62880 | Gi/o | guanine nucleotide binding protein (G protein), beta 2(Gnb2) | pre | 0.594849 | 0.004790908 |
| P29387 | Gi/o | guanine nucleotide binding protein (G protein), beta 4(Gnb4) | pre | ++ | - |
| P62881 | Gi/o | guanine nucleotide binding protein (G protein), beta 5(Gnb5) | pre | 1.771424 | 0.001745108 |
| P63250 | GIRK | potassium inwardly-rectifying channel, subfamily J, member 3(Kcnj3) | pre | ++ | - |
| P43006 | EAAT2 | solute carrier family 1 (glial high affinity glutamate transporter), member 2(Slc1a2) | glia, pre | 1.709416 | 0.000249466 |
| P56564 | EAAT1 | solute carrier family 1 (glial high affinity glutamate transporter), member 3(Slc1a3) | glia | 2.429898 | 0.00117093 |
| Q3TXX4 | VGLU1 | solute carrier family 17 (sodium-dependent inorganic phosphate cotransporter), member 7(Slc17a7) | pre | 4.083932 | 0.000464031 |
| Q62108 | **PSD95/SAP90** | discs, large homolog 4 (Drosophila)(Dlg4) | post | 2.017672 | 0.000368283 |
| P23818 | **GRIA1** | glutamate receptor, ionotropic, AMPA1 (alpha 1)(Gria1) | post | 1.543031 | 0.006536957 |
| P23819 | **GRIA2** | glutamate receptor, ionotropic, AMPA2 (alpha 2)(Gria2) | post | 1.877931 | 0.001499652 |
| Q9Z2W8 | **GRIA4** | glutamate receptor, ionotropic, AMPA4 (alpha 4)(Gria4) | post | ++ |  |
| P35438 | **GRIN1** | glutamate receptor, ionotropic, NMDA1 (zeta 1)(Grin1) | post | 2.143671 | 0.00155659 |
| P35436 | **GRIN2A** | glutamate receptor, ionotropic, NMDA2A (epsilon 1)(Grin2a) | post | 1.99029 | 0.006781272 |
| P39087 | KA | glutamate receptor, ionotropic, kainate 2 (beta 2)(Grik2) | both | ++ | - |
| P97772 | **GRM1** | glutamate receptor, metabotropic 1(Grm1) | post | ++ |  |
| Q9QYS2 | GRM3 | glutamate receptor, metabotropic 3(Grm3) | pre | 1.97833 | 0.000131319 |

Pre indicates a presynaptic location; post indicates a postsynaptic location; both represent presynaptic and postsynaptic locations; glia indicate glial cells.

The *P* value was derived from Student’s *t*-test with significance accepted at 0.01.

**Supplementary Table 5. Fourteen differentially expressed genes identified by RNA-Seq analysis**

| **ID** | **Gene Name** | **Gene Description** | **Locus** | **log_2_FC** | ***P* value** | **UP/DOWN** |
| --- | --- | --- | --- | --- | --- | --- |
| ENSMUSG00000072966 | Gprasp2 | G_protein_coupled_receptor_associated_sorting_protein_2 | X:135839034-135844730 | 1.331642 | 1.56E-21 | UP |
| ENSMUSG00000075224 | Lrrc55 | leucine_rich_repeat_containing_55 | 2:85162334-85196699 | -1.1972 | 1.41E-14 | DOWN |
| ENSMUSG00000094365 | Gm21982 | predicted_gene_21982 | 7:30641001-30664937 | 6.63687 | 6.47E-06 | UP |
| ENSMUSG00000051390 | Zbtb22 | zinc_finger_and_BTB_domain_containing_22 | 17:33915904-33919324 | -1.52893 | 0.00013 | DOWN |
| ENSMUSG00000037872 | Ackr1 | atypical_chemokine_receptor_1_Duffy_blood_group_ | 1:173331886-173333750 | 2.726951 | 0.000132 | UP |
| ENSMUSG00000038729 | Akap2 | A_kinase_PRKA_anchor_protein_2 | 4:57717657-57896982 | 1.148645 | 0.000382 | UP |
| ENSMUSG00000040195 | Tmem194 | transmembrane_protein_194 | 10:127666901-127701049 | 1.20784 | 0.000418 | UP |
| ENSMUSG00000092412 | Gm20507 | predicted_gene_20507 | 17:33630759-33645705 | Inf | 0.00064 | UP |
| ENSMUSG00000045679 | Pqlc3 | PQ_loop_repeat_containing | 12:16988648-17000408 | -1.06442 | 0.000837 | DOWN |
| ENSMUSG00000046387 | Pcdhb17 | protocadherin_beta_17 | 18:37484795-37489454 | Inf | 0.001782 | UP |
| ENSMUSG00000096929 | A330023F24Rik | RIKEN_cDNA_A330023F24_gene | 1:194976375-195037904 | 2.028582 | 0.002705 | UP |
| ENSMUSG00000050761 | Gp1bb | glycoprotein_Ib_beta_polypeptide | 16:18620319-18622403 | -3.41668 | 0.006946 | DOWN |
| ENSMUSG00000064363 | mt-Nd4 | mitochondrially_encoded_NADH_dehydrogenase_4 | MT:10167-11544 | Inf | 0.018415 | UP |
| ENSMUSG00000095538 | Gm21983 | predicted_gene_21983 | 7:27168430-27181086 | Inf | 0.019578 | UP |

UP and DOWN represent upregulated and downregulated differentially expressed genes (DEGs) in R882H-KI HOM mice, respectively, for an FDR-corrected *P* value threshold less than 0.05.

**Supplementary Table 6. Behavioral phenotypes of KI model mice with a missense mutation of a specific gene identified in ASD cases**

| **Missense mutation in ASD cases**  **(in mouse model)** | **Genotype** | **Behavioral Phenotypes** | | | | | | | | | | **References** |
| --- | --- | --- | --- | --- | --- | --- | --- | --- | --- | --- | --- | --- |
|  |  | **Social behavior** | | **Repetitive behavior (RRB)** | | | | **Locomotion** | | **Anxiety** | |  |
|  |  |  |  | **Lower-order RRB** | | **Higher-order RRB** | |  |  |  |  |  |
| *ATP1A3*  I1810N  (I1810N) | HET | Interaction↓ | 3-C | NA | NA | NA | NA | NA | NA | NA | NA | Kirshenbaum et al., 2016 ([55](#_ENREF_55)) |
|  |  | Novelty↓ | 3-C |  |  |  |  |  |  |  |  |  |
|  | HOM | NA | NA | NA | NA | NA | NA | NA | NA | NA | NA |  |
|  |  |  |  |  |  |  |  |  |  |  |  |  |
| *CAMK2A*  E183V  (E183V) | HET | Interaction - | 3-C | — | OF/3-C | - | MB | - | OF | ↑ | OF | Stephenson et al., 2017 ([56](#_ENREF_56)) |
|  |  | Novelty↓ | 3-C |  |  |  |  |  |  |  |  |  |
|  | HOM | Interaction↓ | 3-C | Jumping↑  Rearing↑ | OF | - | MB | ↑ | OF | ↑ | OF |  |
|  |  | Novelty - | 3-C | Circling↑ | 3-C |  |  |  |  |  |  |  |
|  |  |  |  |  |  |  |  |  |  |  |  |  |
| *IQSEC2*  A350V  (A350V) | HET | NA | NA | NA | NA | NA | NA | NA | NA | NA | NA | Rogers et al., 2019 ([57](#_ENREF_57)) |
|  | HOM | Interaction - | 3-C | NA | NA | NA | NA | ↑ | OF | - | OF |  |
|  |  | Novelty - | 3-C |  |  |  |  |  |  |  |  |  |
|  |  |  |  |  |  |  |  |  |  |  |  |  |
| *NLGN1*  P89L  (P89L) | HET | Interaction↓ | 3-C/RSI | Self-Grooming - | Cage | - | MB | - | OF | - | EPM | Nakanishi et al., 2017 ([58](#_ENREF_58)) |
|  | HOM | Interaction - | 3-C/RSI | Self-Grooming - | Cage | - | MB | - | OF | - | EPM |  |
|  |  |  |  |  |  |  |  |  |  |  |  |  |
| *NL3*  R451C  (R451C)^*^ | X-linked | Interaction - | 3-C/RSI | Stereotypic Movements↑ | OF | ↑ | RONC | ↑ | OF/EPM | - | OF/EPM/LD | Tabuchi et al., 2007; Etherton et al., 2011; Rothwell et al., 2014; Jaramillo et al., 2014; Burrows et al., 2015; Cao et al., 2018 ([16](#_ENREF_16), [20](#_ENREF_20), [59-62](#_ENREF_59)) |
|  |  | Novelty↓ | 3-C | Motor Routine↑ | AR |  |  |  |  |  |  |  |
|  |  |  |  |  |  |  |  |  |  |  |  |  |
| *NR2F1*  R112K  (R109K) | HET | Interaction↓ | 3-C | Self-Grooming↑ | Cage | ↑ | YM | - | OF | ↑ | OF/EPM/LD | Zhang et al., 2020 ([63](#_ENREF_63)) |
|  |  | Novelty↓ | 3-C |  |  |  |  |  |  |  |  |  |
|  | HOM | NA | NA | NA | NA | NA | NA | NA | NA | NA | NA |  |
|  |  |  |  |  |  |  |  |  |  |  |  |  |
| *POGZ*  Q1042R  (Q1038R) | HET | Interaction↓ | RSI | Self-Grooming↑ | Cage | NA | NA | - | OF | ↓ | OF | Matsumura et al., 2020 ([64](#_ENREF_64)) |
|  |  |  |  |  |  |  |  |  |  | - | LD |  |
|  | HOM | NA | NA | NA | NA | NA | NA | NA | NA | NA | NA |  |
|  |  |  |  |  |  |  |  |  |  |  |  |  |
| *SHANK3*  S685I  (S685I) | HET | Interaction - | 3-C | Self-Grooming - | Cage | - | MB | - | OF | - | OF/EPM | Wang et al., 2019 ([65](#_ENREF_65)) |
|  | HOM | Interaction - | 3-C | Self-Grooming - | Cage | - | MB | - | OF | - | OF/EPM |  |
|  |  |  |  |  |  |  |  |  |  |  |  |  |
| *SHANK3*  Q321R  (Q321R) | HET | Interaction - | 3-C | Self-Grooming↑  Digging↓ | Cage | NA | NA | - | OF/Cage | - | OF/EPM | Yoo et al., 2019 ([66](#_ENREF_66)) |
|  |  |  |  | Self-Grooming - | Laboras |  |  |  |  | ↓ | LD |  |
|  | HOM | Interaction - | 3-C | Self-Grooming↑  Digging↓ | Cage | NA | NA | - | OF/Cage | - | OF |  |
|  |  |  |  | Self-Grooming - | Laboras |  |  |  |  | ↓ | EPM/LD |  |
|  |  |  |  |  |  |  |  |  |  |  |  |  |
| *SLC6A3*  G56A  (G56A) | HET | NA | NA | NA | NA | NA | NA | NA | NA | NA | NA | Veenstra-VanderWeele et al., 2012 ([67](#_ENREF_67)) |
|  | HOM | Interaction - | 3-C | Hanging↑  Awaken/Sleep –  Chew/Eat/Drink –  Rearing –  Self-Grooming –  Remaining low –  Sniffing –  Stretching –  Twitching –  Walking – | Cage | - | MB | - | OF | - | EPM |  |
|  |  |  |  |  |  |  |  |  |  |  |  |  |
| *SLC6A3*  T356M  (T356M) | HET | - | 3-C | - | Cage/AR | - | MB | - | OF | - | EZM | DiCarlo et al., 2019 ([68](#_ENREF_68)) |
|  | HOM | Interaction - | 3-C | Rearing↑  Self-Grooming –  Digging –  Jumping –  Climbing –  Tail Flick - | Cage | ↓ | MB | ↑ | OF | - | EZM |  |
|  |  |  |  | Motor Routine↑ | AR |  |  |  |  |  |  |  |
|  |  |  |  |  |  |  |  |  |  |  |  |  |
| *TBR1*  K228E  (K228E) | HET | Interaction↓ | RSI | Self-Grooming↑  Rearing↑ | Laboras | NA | NA | - | OF | ↓ | OF | Yook et al., 2019 ([69](#_ENREF_69)) |
|  |  | Interaction - | 3-C |  |  |  |  |  |  | ↑ | EPM/LD |  |
|  |  | Novelty - | 3-C |  |  |  |  |  |  |  |  |  |
|  | HOM | NA | NA | NA | NA | NA | NA | NA | NA | NA | NA |  |
|  |  |  |  |  |  |  |  |  |  |  |  |  |
| *SHANK1*  R874H  (R882H) | HET | Interaction - | 3-C | NA | NA | ↓ | MB | - | OF | - | OF/LD | This study |
|  |  | Novelty↓ | 3-C |  |  |  |  |  |  |  |  |  |
|  | HOM | Interaction - | 3-C | NA | NA | ↓ | MB | - | OF | - | OF/LD |  |
|  |  | Novelty↓ | 3-C |  |  |  |  |  |  |  |  |  |

For a specific phenotype, the index is listed on the left, with the corresponding test or assay listed on the right. An upward arrow represents an increase, a downward arrow represents a decrease, and a hyphen represents no change.

3-C, three-chamber social test; RSI, reciprocal social interaction test; Cage, cage observation test; Laboras, Laboras cage observation test; MB, marble-burying test; AR, accelerating rotarod test; OF, open field test; EPM, elevated plus maze test; EZM, elevated zero maze test; LD, light-dark exploration test; RONC, repetitive object novel contact test; YM, Y-maze test; NA, not analyzed.

*consistent findings in this line summarized from multiple studies.

**Supplementary Table 7. DNA sequences of primer pairs used for rare mutation screening in *SHANK1***

| **Sequence Name** | **Forward primer/Reverse primer (5’-3’)** | **Purpose** |
| --- | --- | --- |
| SHANK1-PCR1 | GCCTCCTTCCTGCCTATCTT/TGGGGAAGAGATAGGGGCTA | Mutation screening |
| SHANK1-PCR2 | CTCAGCTCCTGGCTCACTTC/AGGCCACTTAGCTGGTCAGA | Mutation screening |
| SHANK1-PCR3 | ACCCAGCACTGTCGGAAAAG/ AGCATCCCAAGTTAAGCGGG | Mutation screening |
| SHANK1-PCR4 | CTGCCTTGCTCCTGACTGT/CAAAGGGAAAAGGAGAGAGG | Mutation screening |
| SHANK1-PCR5 | TGGAGGCATCTACCCAAGTC/TTATTCTCACCCCCATGCTC | Mutation screening |
| SHANK1-PCR6 | GATGCGGTGAACGATAGGAT/GTTGTCTGCCCTCTGCTAGG | Mutation screening |
| SHANK1-PCR7 | GCGGGTTTAGGGTGGAGTAT/CCCAGGTTCTCTGTGCAGTC | Mutation screening |
| SHANK1-PCR8 | CCCCTCCCTTTCTCATCTCT/TTGCTTTACCCCAAGGTGAC | Mutation screening |
| SHANK1-PCR9 | AAATGCCCTCTTCCTTGGAG/CACTTCACTGCCTCCTGACA | Mutation screening |
| SHANK1-PCR10 | GCCCCTTCCTCAGATGTTTA/GGGCTTAGAGGTGATGGAAA | Mutation screening |
| SHANK1-PCR11 | CTGGGGTAGCCAGAGACAAC/AGCCCCACAGTCATCATAGC | Mutation screening |
| SHANK1-PCR12 | GGGGACAGCCTCTGTGTCT/GTGAATCATGAGGGGGTCTG | Mutation screening |
| SHANK1-PCR13 | ACCCCTCCCCTCGTCTACT/GAACTCAAGGGATGGTCCAG | Mutation screening |
| SHANK1-PCR14 | GAGTGGTGAGTGGGCACAG/ACACAATCTCCCAGCCCAGT | Mutation screening |
| SHANK1-PCR15 | GGGAGATTGTGTCTCCAAGC/TTGAGGGAAAGGGGATAAGG | Mutation screening |
| SHANK1-PCR16 | CCTTCCACCGTCTTCACACT/GGGCTTTAGCTCACTGCATC | Mutation screening |
| SHANK1-PCR17 | CTCACCTTCCTTGCGGTTT/CCCACTGTGGTACAGGCTCT | Mutation screening |
| SHANK1-PCR18 | CCCTACAGCACACCTCCAGT/CTTCCACCTTGGTCTGCTTG | Mutation screening |
| SHANK1-PCR19 | CGGCAGAAGATGACAGACCT/GGCAGCTGGAAATAGCGTAG | Mutation screening |
| SHANK1-PCR20 | CGGCTCTACGCTATTTCCAG/GAACTGGCTCGTGAAGTCCA | Mutation screening |
| SHANK1-PCR21 | CCCTCGGAGAAGAACTCCAT/GCAGGAAGCTGGTGAAGG | Mutation screening |
| SHANK1-PCR22 | ACTCCAAATCCATCGACGAG/ACCTCCAGGGCTTGCTTACT | Mutation screening |
| SHANK1-PCR23 | AGCCAGGAGAAGTCCCTTCC/TAGGATGTCAGGCTGGATGC | Mutation screening |
| SHANK1-PCR24 | AACAGCTTCGAAAAGCCAGA/ATGAGCTGAGGAGGGTATGG | Mutation screening |
| SHANK1-PCR25 | ACCTATGTGGCCTACCTGGA/GACCCTAAAGGGACACAGCC | Mutation screening |
| SHANK1-PCR26 | CGTCTCTCCCCCTACCCTTA/TGTCAAAGAGGCCTGGGTAG | Mutation screening |
| SHANK1-PCR27 | CCCCCTAAATCCCTGTCCT/ACGTCGAACTTGGTCCAGAA | Mutation screening |
| SHANK1-PCR28 | CTCTACCCAGGCCTCTTTGA/CCCTTCAGTGAGGTGCAAAT | Mutation screening |

**Supplementary Table 8. DNA sequences used for plasmid construction and for generation or genotyping of the knock-in mice**

| **Sequence Name** | **Forward primer/Reverse primer or sequence (5’-3’)** | **Purpose** |
| --- | --- | --- |
| Shank1-A612V-F/R | GTAAACCGTTCCCAGGAGGGAAGACAAG/ CACCTCTTCTAGGCAGTCAGAAGGGAACC | Plasmid Mutagenesis |
| Shank1-R874H-F/R | CATCCTTCTTTCCTGCCTCCTGGACC/ GTCATAACTTGGCTGGGAGCGGTGG | Plasmid Mutagenesis |
| Shank1-P1806L-F/R | CTCTCAACGGCAGGTGTGGCAGGG/ TCCAGGGCAAGCACTCAAGGCCAG | Plasmid Mutagenesis |
| Shank1-D1926N-F/R | AACGACTCCCAGACCTCTCTCCTCTCC/ AGAGAGCCTCTGCCGCTGCAGGGAGGA | Plasmid Mutagenesis |
| Shank1-G2026N-F/R | AGACCCATCTACCCGGGCCTCTTCGAC/ TGAAGGCAGGATGGGCAAAGAGGAAGGCC | Plasmid Mutagenesis |
| Shank1-G2037D-F/R | GACTCTCCAACCGGAGGGGCGGGAG/ GACTCTCCAACCGGAGGGGCGGGAGGCT | Plasmid Mutagenesis |
| sgRNA | GCTCCCAGCCAAGTTATGAC | Generate knock-in mice |
| ssODN | CTGCCCCGAAGTCGAGCTTTGATCCTCACCACCGCTCCCAGCCAAGTTATGACcacCCTTCTTTCCTGCCTCCCGGACCTGGCCTTATGCTCCGGCAGAAATCTATCGGTATGTCC | Generate knock-in mice |
| GT-882 | GAGTGTCAATGGGACCTGGG/ CTGGGCTACGGTCCTTTCTG | Genotyping in mice |

**References**

1. Zheng H, Tao R, Zhang J, Zhang J, Wang S, Yang Z, et al. Development and validation of a novel SiFaSTR(TM) 23-plex system. Electrophoresis. 2019;40(20):2644-54.

2. Genomes Project C, Abecasis GR, Auton A, Brooks LD, DePristo MA, Durbin RM, et al. An integrated map of genetic variation from 1,092 human genomes. Nature. 2012;491(7422):56-65.

3. Lek M, Karczewski KJ, Minikel EV, Samocha KE, Banks E, Fennell T, et al. Analysis of protein-coding genetic variation in 60,706 humans. Nature. 2016;536(7616):285-91.

4. Karczewski KJ, Francioli LC, Tiao G, Cummings BB, Alfoldi J, Wang QB, et al. The mutational constraint spectrum quantified from variation in 141,456 humans. Nature. 2020;581(7809):434-43.

5. Liu S, Huang S, Chen F, Zhao L, Yuan Y, Francis SS, et al. Genomic Analyses from Non-invasive Prenatal Testing Reveal Genetic Associations, Patterns of Viral Infections, and Chinese Population History. Cell. 2018;175(2):347-59 e14.

6. Ramani R, Krumholz K, Huang YF, Siepel A. PhastWeb: a web interface for evolutionary conservation scoring of multiple sequence alignments using phastCons and phyloP. Bioinformatics. 2019;35(13):2320-2.

7. Kumar P, Henikoff S, Ng PC. Predicting the effects of coding non-synonymous variants on protein function using the SIFT algorithm. Nat Protoc. 2009;4(7):1073-82.

8. Adzhubei IA, Schmidt S, Peshkin L, Ramensky VE, Gerasimova A, Bork P, et al. A method and server for predicting damaging missense mutations. Nature methods. 2010;7(4):248-9.

9. Schwarz JM, Cooper DN, Schuelke M, Seelow D. MutationTaster2: mutation prediction for the deep-sequencing age. Nature methods. 2014;11(4):361-2.

10. Kircher M, Witten DM, Jain P, O'Roak BJ, Cooper GM, Shendure J. A general framework for estimating the relative pathogenicity of human genetic variants. Nature genetics. 2014;46(3):310-5.

11. Quang D, Chen Y, Xie X. DANN: a deep learning approach for annotating the pathogenicity of genetic variants. Bioinformatics. 2015;31(5):761-3.

12. Sala C, Piech V, Wilson NR, Passafaro M, Liu G, Sheng M. Regulation of dendritic spine morphology and synaptic function by Shank and Homer. Neuron. 2001;31(1):115-30.

13. Liu W, Li K, Bai D, Yin J, Tang Y, Chi F, et al. Dosage effects of ZP2 and ZP3 heterozygous mutations cause human infertility. Human genetics. 2017;136(8):975-85.

14. Torres L, Danver J, Ji K, Miyauchi JT, Chen D, Anderson ME, et al. Dynamic microglial modulation of spatial learning and social behavior. Brain, behavior, and immunity. 2016;55:6-16.

15. Thomas A, Burant A, Bui N, Graham D, Yuva-Paylor LA, Paylor R. Marble burying reflects a repetitive and perseverative behavior more than novelty-induced anxiety. Psychopharmacology. 2009;204(2):361-73.

16. Etherton M, Foldy C, Sharma M, Tabuchi K, Liu XR, Shamloo M, et al. Autism-linked neuroligin-3 R451C mutation differentially alters hippocampal and cortical synaptic function. P Natl Acad Sci USA. 2011;108(33):13764-9.

17. Silverman JL, Turner SM, Barkan CL, Tolu SS, Saxena R, Hung AY, et al. Sociability and motor functions in Shank1 mutant mice. Brain Res. 2011;1380:120-37.

18. Angoa-Perez M, Kane MJ, Briggs DI, Francescutti DM, Kuhn DM. Marble Burying and Nestlet Shredding as Tests of Repetitive, Compulsive-like Behaviors in Mice. Jove-J Vis Exp. 2013(82):e50978.

19. Houle K, Abdi M, Clabough EBD. Acute ethanol exposure during late mouse neurodevelopment results in long-term deficits in memory retrieval, but not in social responsiveness. Brain Behav. 2017;7(4):e00636.

20. Cao W, Lin S, Xia QQ, Du YL, Yang Q, Zhang MY, et al. Gamma Oscillation Dysfunction in mPFC Leads to Social Deficits in Neuroligin 3 R451C Knockin Mice (vol 97, pg 1253, 2018). Neuron. 2018;98(3):670-.

21. Yang M, Silverman JL, Crawley JN. Automated three-chambered social approach task for mice. Current protocols in neuroscience. 2011;Chapter 8:Unit 8 26.

22. Komada M, Takao K, Miyakawa T. Elevated plus maze for mice. Journal of visualized experiments : JoVE. 2008(22).

23. Bevins RA, Besheer J. Object recognition in rats and mice: a one-trial non-matching-to-sample learning task to study 'recognition memory'. Nat Protoc. 2006;1(3):1306-11.

24. Lueptow LM. Novel Object Recognition Test for the Investigation of Learning and Memory in Mice. Journal of visualized experiments : JoVE. 2017(126).

25. Ashburner J, Friston KJ. Why voxel-based morphometry should be used. Neuroimage. 2001;14(6):1238-43.

26. Ullmann JFP, Watson C, Janke AL, Kurniawan ND, Reutens DC. A segmentation protocol and MRI atlas of the C57BL/6J mouse neocortex. Neuroimage. 2013;78:196-203.

27. Alberi L, Liu SX, Wang Y, Badie R, Smith-Hicks C, Wu J, et al. Activity-Induced Notch Signaling in Neurons Requires Arc/Arg3.1 and Is Essential for Synaptic Plasticity in Hippocampal Networks. Neuron. 2011;69(3):437-44.

28. Zhao QR, Lu JM, Yao JJ, Zhang ZY, Ling C, Mei YA. Neuritin reverses deficits in murine novel object associative recognition memory caused by exposure to extremely low-frequency (50 Hz) electromagnetic fields. Sci Rep-Uk. 2015;5:11768.

29. Paxinos G, Franklin KBJ, Franklin KBJ. The mouse brain in stereotaxic coordinates. 2nd ed. San Diego: Academic Press; 2001.

30. Peca J, Feliciano C, Ting JT, Wang W, Wells MF, Venkatraman TN, et al. Shank3 mutant mice display autistic-like behaviours and striatal dysfunction. Nature. 2011;472(7344):437-42.

31. Schmeisser MJ, Ey E, Wegener S, Bockmann J, Stempel AV, Kuebler A, et al. Autistic-like behaviours and hyperactivity in mice lacking ProSAP1/Shank2. Nature. 2012;486(7402):256-60.

32. Hung AY, Futai K, Sala C, Valtschanoff JG, Ryu J, Woodworth MA, et al. Smaller dendritic spines, weaker synaptic transmission, but enhanced spatial learning in mice lacking Shank1. The Journal of neuroscience : the official journal of the Society for Neuroscience. 2008;28(7):1697-708.

33. Harris KM, Weinberg RJ. Ultrastructure of synapses in the mammalian brain. Cold Spring Harbor perspectives in biology. 2012;4(5):a005587.

34. Jawaid S, Kidd GJ, Wang J, Swetlik C, Dutta R, Trapp BD. Alterations in CA1 hippocampal synapses in a mouse model of fragile X syndrome. Glia. 2018;66(4):789-800.

35. Huang DW, Sherman BT, Lempicki RA. Systematic and integrative analysis of large gene lists using DAVID bioinformatics resources. Nat Protoc. 2009;4(1):44-57.

36. Huang DW, Sherman BT, Lempicki RA. Bioinformatics enrichment tools: paths toward the comprehensive functional analysis of large gene lists. Nucleic Acids Res. 2009;37(1):1-13.

37. Bermejo MK, Milenkovic M, Salahpour A, Ramsey AJ. Preparation of Synaptic Plasma Membrane and Postsynaptic Density Proteins Using a Discontinuous Sucrose Gradient. Jove-J Vis Exp. 2014(91):e51896.

38. Wang X, Bey AL, Katz BM, Badea A, Kim N, David LK, et al. Altered mGluR5-Homer scaffolds and corticostriatal connectivity in a Shank3 complete knockout model of autism. Nat Commun. 2016;7:11459.

39. Distler U, Schmeisser MJ, Pelosi A, Reim D, Kuharev J, Weiczner R, et al. In-depth protein profiling of the postsynaptic density from mouse hippocampus using data-independent acquisition proteomics. Proteomics. 2014;14(21-22):2607-13.

40. Chen SS, Luo Y, Ding GD, Xu FS. Comparative analysis of Brassica napus plasma membrane proteins under phosphorus deficiency using label-free and MaxQuant-based proteomics approaches. J Proteomics. 2016;133:144-52.

41. Reim D, Distler U, Halbedl S, Verpelli C, Sala C, Bockmann J, et al. Proteomic Analysis of Post-synaptic Density Fractions from Shank3 Mutant Mice Reveals Brain Region Specific Changes Relevant to Autism Spectrum Disorder. Frontiers in molecular neuroscience. 2017;10:26.

42. Wisniewski JR, Zougman A, Nagaraj N, Mann M. Universal sample preparation method for proteome analysis. Nature methods. 2009;6(5):359-U60.

43. Carlin RK, Grab DJ, Cohen RS, Siekevitz P. Isolation and Characterization of Postsynaptic Densities from Various Brain-Regions - Enrichment of Different Types of Postsynaptic Densities. J Cell Biol. 1980;86(3):831-43.

44. Wang J, Ming H, Chen R, Ju JM, Peng WD, Zhang GX, et al. CIH-induced neurocognitive impairments are associated with hippocampal Ca(2+) overload, apoptosis, and dephosphorylation of ERK1/2 and CREB that are mediated by overactivation of NMDARs. Brain Res. 2015;1625:64-72.

45. Schoepp DD, Jane DE, Monn JA. Pharmacological agents acting at subtypes of metabotropic glutamate receptors. Neuropharmacology. 1999;38(10):1431-76.

46. Mao LM, Wang JQ. Glutamate cascade to cAMP response element-binding protein phosphorylation in cultured striatal neurons through calcium-coupled group I metabotropic glutamate receptors. Mol Pharmacol. 2002;62(3):473-84.

47. Thomson SR, Seo SS, Barnes SA, Louros SR, Muscas M, Dando O, et al. Cell-Type-Specific Translation Profiling Reveals a Novel Strategy for Treating Fragile X Syndrome. Neuron. 2017;95(3):550-63.

48. Detillion CE, Craft TK, Glasper ER, Prendergast BJ, DeVries AC. Social facilitation of wound healing. Psychoneuroendocrinology. 2004;29(8):1004-11.

49. Bicks LK, Yamamuro K, Flanigan ME, Kim JM, Kato D, Lucas EK, et al. Prefrontal parvalbumin interneurons require juvenile social experience to establish adult social behavior. Nat Commun. 2020;11(1):1003.

50. Sato D, Lionel AC, Leblond CS, Prasad A, Pinto D, Walker S, et al. SHANK1 Deletions in Males with Autism Spectrum Disorder. American journal of human genetics. 2012;90(5):879-87.

51. Leblond CS, Nava C, Polge A, Gauthier J, Huguet G, Lumbroso S, et al. Meta-analysis of SHANK Mutations in Autism Spectrum Disorders: a gradient of severity in cognitive impairments. PLoS genetics. 2014;10(9):e1004580.

52. Krumm N, Turner TN, Baker C, Vives L, Mohajeri K, Witherspoon K, et al. Excess of rare, inherited truncating mutations in autism. Nature genetics. 2015;47(6):582-8.

53. Wang T, Guo H, Xiong B, Stessman HAF, Wu H, Coe BP, et al. De novo genic mutations among a Chinese autism spectrum disorder cohort. Nature Communications. 2016;7:13316.

54. Monies D, Abouelhoda M, Assoum M, Moghrabi N, Rafiullah R, Almontashiri N, et al. Lessons Learned from Large-Scale, First-Tier Clinical Exome Sequencing in a Highly Consanguineous Population. American journal of human genetics. 2019;105(4):879.

55. Kirshenbaum GS, Idris NF, Dachtler J, Roder JC, Clapcote SJ. Deficits in social behavioral tests in a mouse model of alternating hemiplegia of childhood. Journal of neurogenetics. 2016;30(1):42-9.

56. Stephenson JR, Wang X, Perfitt TL, Parrish WP, Shonesy BC, Marks CR, et al. A Novel Human CAMK2A Mutation Disrupts Dendritic Morphology and Synaptic Transmission, and Causes ASD-Related Behaviors. The Journal of neuroscience : the official journal of the Society for Neuroscience. 2017;37(8):2216-33.

57. Rogers EJ, Jada R, Schragenheim-Rozales K, Sah M, Cortes M, Florence M, et al. An IQSEC2 Mutation Associated With Intellectual Disability and Autism Results in Decreased Surface AMPA Receptors. Frontiers in molecular neuroscience. 2019;12:43.

58. Nakanishi M, Nomura J, Ji X, Tamada K, Arai T, Takahashi E, et al. Functional significance of rare neuroligin 1 variants found in autism. PLoS genetics. 2017;13(8):e1006940.

59. Tabuchi K, Blundell J, Etherton MR, Hammer RE, Liu XR, Powell CM, et al. Neuroligin-3 mutation implicated in autism increases inhibitory synaptic transmission in mice. Science. 2007;318(5847):71-6.

60. Rothwell PE, Fuccillo MV, Maxeiner S, Hayton SJ, Gokce O, Lim BK, et al. Autism-Associated Neuroligin-3 Mutations Commonly Impair Striatal Circuits to Boost Repetitive Behaviors. Cell. 2014;158(1):198-212.

61. Jaramillo TC, Liu SN, Pettersen A, Birnbaum SG, Powell CM. Autism-Related Neuroligin-3 Mutation Alters Social Behavior and Spatial Learning. Autism Research. 2014;7(2):264-72.

62. Burrows EL, Laskaris L, Koyama L, Churilov L, Bornstein JC, Hill-Yardin EL, et al. A neuroligin-3 mutation implicated in autism causes abnormal aggression and increases repetitive behavior in mice. Molecular Autism. 2015;6:62.

63. Zhang K, Yu F, Zhu J, Han S, Chen JH, Wu XY, et al. Imbalance of Excitatory/Inhibitory Neuron Differentiation in Neurodevelopmental Disorders with an NR2F1 Point Mutation. Cell Rep. 2020;31(3):107521.

64. Matsumura K, Seiriki K, Okada S, Nagase M, Ayabe S, Yamada I, et al. Pathogenic POGZ mutation causes impaired cortical development and reversible autism-like phenotypes. Nature Communications. 2020;11(1):859.

65. Wang L, Pang K, Han K, Adamski CJ, Wang W, He L, et al. An autism-linked missense mutation in SHANK3 reveals the modularity of Shank3 function. Molecular psychiatry. 2020;25(10):2534-55.

66. Yoo YE, Yoo T, Lee S, Lee J, Kim D, Han HM, et al. Shank3 Mice Carrying the Human Q321R Mutation Display Enhanced Self-Grooming, Abnormal Electroencephalogram Patterns, and Suppressed Neuronal Excitability and Seizure Susceptibility. Frontiers in molecular neuroscience. 2019;12:155.

67. Veenstra-VanderWeele J, Muller CL, Iwamoto H, Sauer JE, Owens WA, Shah CR, et al. Autism gene variant causes hyperserotonemia, serotonin receptor hypersensitivity, social impairment and repetitive behavior. P Natl Acad Sci USA. 2012;109(14):5469-74.

68. DiCarlo GE, Aguilar JI, Matthies HJ, Harrison FE, Bundschuh KE, West A, et al. Autism-linked dopamine transporter mutation alters striatal dopamine neurotransmission and dopamine-dependent behaviors. J Clin Invest. 2019;129(8):3407-19.

69. Yook C, Kim K, Kim D, Kang H, Kim SG, Kim E, et al. A TBR1-K228E Mutation Induces Tbr1 Upregulation, Altered Cortical Distribution of Interneurons, Increased Inhibitory Synaptic Transmission, and Autistic-Like Behavioral Deficits in Mice. Frontiers in molecular neuroscience. 2019;12:241.
